# Skin microbiota during metamorphosis of *Quasipaa spinosa*: guidance for maintaining mucosal symbiotic microbial flora homeostasis in early life of frogs

**DOI:** 10.3389/fmicb.2024.1453617

**Published:** 2024-11-14

**Authors:** Jinliang Hou, Yu Tan, Yanfei Huang, Hong Li, Deliang Li, Xinhua Liu, Junhua Li, Yazhou Hu, Jianguo Xiang

**Affiliations:** ^1^Fisheries College, Hunan Agricultural University, Changsha, China; ^2^Xiangtan Animal Husbandry and Aquatic Technology Research and Promotion Center, Xiangtan, China

**Keywords:** developmental stage, *Quasipaa spinosa*, cutaneous bacterial communities, 16S amplicon sequencing, co-occurrence network

## Abstract

The skin microbiota plays an essential role in helping the host adapt to different environments and maintain health. By examining the characteristics of amphibian skin flora alongside ontogenetic traits, we can gain insights into the adaptation mechanisms of amphibian skin flora to environmental changes during development. In this study, we analyzed the skin microbiota of *Quasipaa spinosa* during metamorphosis using Illumina sequencing. Venn diagrams and UpSet analysis revealed that the LTS (hindlimb tadpoles’ skin, aquatic habitat) and FTS (forelimb tadpoles’ skin, shift from aquatic to amphibious habitats) groups exhibited a higher number of unique amplicon sequence variants (ASVs), while the TS (tadpoles’ skin, aquatic habitat) and LFS (land frogs’ skin, amphibious habitats) groups displayed a lower abundance of ASVs. Diversity analysis indicated similarities in the microorganisms between the LTS and the FTS groups, with higher microbial diversity compared to the TS and the LFS groups. Additionally, microbial co-occurrence network analysis indicated a more stable microecology in the LTS group and FTS group. Proteobacteria, Firmicutes, and Bacteroidota were identified as the dominant phyla, although their relative abundances varied widely among groups. LEfSe (Linear discriminant analysis effect size) showed significant enrichment of beneficial bacteria at various developmental stages, including *Bacteroides*, *Bacillus*, and *Lactobacillus*. Functional prediction analysis shows significant differences in skin microorganism functions across various developmental stages, with a primary focus on metabolic functions. This study provides valuable insights into the compositional dynamics of skin microbiota in *Q. spinosa* at various developmental stages.

## Highlights

- Significant changes in skin microbial diversity in response to changes in developmental and perching habitats.
- Changes in microbial communities at different stages to facilitate host’s adaptation to the changes stemming from both development and environment.
- Several sensitive indicator taxa were identified for predicting host health trends.
- Critical stages determining the success of metamorphic development were identified: forelimb tadpoles to landing frogs.

## 1 Introduction

The skin forms a protective barrier against the external environment and is the first line of defense against external aggressors (Kueneman et al., 2014). While the skin’s barrier function is critically dependent on its colonizing microbiota, which provides protection from pathogens, modulates immune responses, and enhances the epithelium (Harris-Tryon and Grice, 2022; Hu et al., 2021). The skin microbiota of healthy individuals maintains a stable and dynamic equilibrium (Wei et al., 2023). Studies have shown that a number of skin diseases are closely associated with altered microbial status, and these changes are also referred to as microbial dysbiosis (Christensen and Bruggemann, 2014). In recent years, the skin microbial community of aquatic organisms has gradually received more and more attention, but most of it has concentrated on disease-related microbes and the effects of purely environmental changes on the microbes (Chen et al., 2022; Fieschi-Meric et al., 2023; Jiang et al., 2022; Mulla and Hernandez-Gomez, 2023; Sadeghi et al., 2023). In contrast, skin microbial communities are affected by a variety of factors and further research is needed to find the key factors that maintain the stability of the flora.

A very special group of organisms is the tailless amphibians. During metamorphosis, amphibians develop specialized habits as they transition from aquatic life to co-inhabitation of water bodies and land (Hou et al., 2022; Tong et al., 2020). This transition presents a serious challenge to their survival, as exposure-induced habitat modification could disrupt the microecological balance of skin surface microorganisms, posing a serious threat to species health (Martinez-Ugalde et al., 2022). The second reason is that during metamorphosis amphibians in larval stages undergo physiological rearrangements (Brown and Cai, 2007), such as the skin of the organism gains a new function: assisted respiration after completing metamorphosis (Martinez-Ugalde et al., 2022), which cause immunosuppression of hormonal signaling in the organism (Rollins-Smith, 1998), and would allow for a smoother colonization by pathogenic bacteria. An increasing number of studies are investigating the infestation of microbial pathogens as a leading cause of amphibian mortality such as the pathogenic microorganisms causing “chytridiomycosis”(Bletz et al., 2018; Muletz-Wolz et al., 2019; Scheele et al., 2019), rotten-skin (Jiang et al., 2022; Xu et al., 2024a), cataracts (Knutie et al., 2017; Xu et al., 2024a) and torticollis (Xu et al., 2024b), which has been responsible for widespread amphibian deaths. Understanding the skin microbial community of healthy amphibians is important for preventing and controlling skin diseases to promote healthy farming.

The Chinese spiny frog (*Quasipaa spinosa*) belongs to the *Dicroglossidae* family and the *Quasipaa* genus, which belongs to the Anura order and Amphibia of chordates (Hou et al., 2023). *Q. spinosa* is an economically important amphibian species with a high nutritional and medicinal value (Hou et al., 2022). However, the vulnerability of the metamorphosis period and the harsh environmental requirements contribute to a high mortality rate. Previous studies have suggested a potential link between changes in gut flora and this phenomenon (Yu et al., 2014). However, the occurrence of disease phenomena like rotten-skin during metamorphosis raises questions about the role of skin microbiota in ensuring healthy survival (Hou et al., 2022). Although some studies have shown that alterations in the skin microflora of Echinodermata can lead to diseases, there is a lack of research on how developmental habitat changes relate to the dynamics of skin microflora.

In this study, skin samples were collected from spiny-breasted frogs at four key points during metamorphosis: complete tadpoles, tadpoles with hindlimbs, tadpoles with forelimbs, and amphibious living frogs. The 16S RNA and Illumina high-throughput sequencing technology were deployed to systematically compare differences in the composition, diversity, and biological function of the skin microbiota during the stages of metamorphosis. Our work sought to (1) elucidate modifications in the composition and diversity of the skin microbiota during the stages of metamorphosis; (2) predict skin microbiota-mediated functional alterations; (3) screen the indicator microorganism taxa that represent the four key stages of health. The study results emphasize the potential relevance of skin microbiota to the developmental stages of host metamorphosis, and highlight how the skin’s symbiotic microbial community responds to physiological and habitat changes caused by development in healthy hosts, with the goal of providing valuable information for healthy culture and improved survival of *Q. spinosa* from a microbial perspective.

## 2 Materials and methods

### 2.1 Sample collection and preservation

The Chinese spiny frogs and tadpoles used in this study were captured from a breeding farm (111°02′40′′ E, 29°47′54′′ N, 150 m asl) in Shimen County, Changde City, Hunan Province, China. The experiment was carried out in a breeding pond for 5 months. The water was cultivated spring water at 19 ± 0.5°C, and its quality was deemed to be first class according to the Environmental Quality Standards for Surface Water in China (GB3838-2002). All frogs and tadpoles shared the same genetic background and were at the different critical stages of metamorphosis. Selective sampling was performed in order to make each stage sample representative. The tadpoles/frogs at a stage were of similar size and developmental period, and all samples were collected from healthy individuals. However, we ensured that all tadpoles were fertilized and hatched at the same time period, and that other external factors, such as the breeding environment, were consistent. Throughout the growth phase, these frogs were given the same feed and feed amount. No antibiotics were used during this experiment. According to Gosner (1960) and Kimball (2016), the developmental stage of the tadpole was determined. The TS group were tadpoles that had just started to feed after demetamorphosis (GS 25). The LTS group were tadpoles that had grown hind limbs but had not yet formed forelimbs and were in the GS 31 ~ GS 37. The FTS group were tadpoles that had already extended their forelimbs (GS 42 ~ GS 45). The LFS group were a developmental stage of frogs that had recently undergone metamorphosis and had not yet consumed food (GS 46). Six samples were collected from each group (Table 1). The weight and length of tadpoles/frogs were recorded at the same time of sampling (Table 1).

**Table 1:** Host information of 24 skin swab samples of *Q. spinosa*.

	Tadpoles	Hindlimb tadpoles	Forelimb tadpoles	Land frogs
Total length (mm)	3.01 ± 0.08	4.96 ± 0.05	5.71 ± 0.08	2.29 ± 0.16
Weight (g)	0.31 ± 0.01	1.12 ± 0.04	1.76 ± 0.10	1.23 ± 0.02
Sample number	6	6	6	6
Feeding state	1	1	0	0

1 represented eating state, and 0 represented fasting state.

Prior to sampling, each spiny frog was rinsed with 1 × PBS (phosphate buffered saline; cytiva, United States). The skin microbiota was harvested using cotton swabs according to a previously described protocol (Costa et al., 2016). Swabs were then transferred into lyophilization tubes and immediately preserved in liquid nitrogen until DNA extraction. To ensure clean samples, the entire sampling process was carried out in ultra-clean bench with aseptic manipulation. To avoid cross contamination between samples, the instruments were replaced with new sterile cotton swabs when sampling different individuals.

### 2.2 DNA extraction, and PCR amplification

The swabs were transferred into sterile centrifuge tubes with 1 × PBS (cytiva, United States), and the liquid was retained for DNA extraction after ultrasonic elution treatment. Total microbial genomic DNA was extracted from each sample with a MgaPure Stool DNA LQ Kit (Magen, Guangzhou, China) according to the manufacturer’s protocol.

Amplification of the V3-V4 regions of the 16S rRNA genes was conducted with universal primers 341F and 805R (Table 2). The KAPA HiFi HotStart ReadyMix PCR kit was used for PCR amplification according to the instructions. The system consisted of 15 μL 2 × KAPA HiFi Mix, 12.5 μL of template, 1 μL of forward primer, 1 μL of reverse primer and then add dd H_2_O to 30 μL, and the reaction conditions were as follows: pre-denaturation at 95°C for 3 min, denaturation at 95°C for 30 s, annealing at 55°C for 30 s, extension at 72°C for 15 s for 25 cycles, and finally extension at 72°C for 5 min. The PCR products were separated and purified from a 1% agarose gel by agarose gel electrophoresis using SanPrep Column DNA Gel Extraction Kit (Sangon Biotech, China) according to the instructions. The recovered products were quantified by Qubit Fluorometer (Thermo Fisher Scientific, United States) and mixed accordingly. Sequencing libraries were prepared using the TruSeq Nano DNA LT Library Prep Kit according to the instructions. The raw reads of skin microbial sequences were deposited in the NCBI SRA database under accession numbers PRJNA1058082.

**Table 2:** Primers used for this study.

Primer name	Nucleotide sequence (5′-3′)	Application
341F	CCTACGGGNGGCWGCAG	Amplification of the V3-V4 regions of the 16S rRNA genes
805R	GACTAHVGGGTATCTAATCC	

### 2.3 Illumina sequencing

Following amplicon level standardization, all samples were submitted to Benagen Technology Company Limited (Wuhan, China) for high-throughput sequencing using the Novaseq platform (Illumina, United States).

### 2.4 Preprocessing of sequencing data

The library containing mixed samples was identified by barcode to obtain single sample sequence data. Low-quality filtering was performed using Trimmomatic v0.39 (Bolger et al., 2014) software with a sliding window size of 20 bp and an average quality score threshold within the window set to greater than 20. The primer sequences were then identified and removed using cutadapt (version 3.4) software to obtain clean sequences without primers (Martin, 2011). The obtained clean reads were filtered, denoised, and merged into amplicon sequence variants (ASVs) using DADA2 (Callahan et al., 2016).

### 2.5 Bioinformatics and statistical analysis

Taxonomic classification of each ASV was assigned against the Silva database (Release138) using QIIME2 (2021.4) (Bokulich et al., 2018; Quast et al., 2013). Venn and UpSet analyses were conducted using TBtools (2.012) to the determine exclusive and shared ASVs among groups. Metrics of alpha-diversity, including richness indexes (ACE (Abundance-based Coverage Estimator) and Chao1), diversity indexes (Shannon), and evenness index (Simpson), were calculated based on vegan (2.6–4) and ggplot2 (3.4.4) package of R, respectively. The significance was performed through one-way analysis of variance (ANOVA) followed by Turkey’s post-hoc multiple comparisons. For beta-diversity analysis, Bray–curtis dissimilarity matrices were calculated based on microeco (1.5.0), and visualized by NMDS (Non-metric multidimensional scaling) using ggplot2 (Liu et al., 2021). Ternary plot was calculated and visualized based on ggtern (3.4.2) package of R. Sequences with the highest abundance of features at the genus level were chosen as representative sequences. Multiple sequence alignment was conducted using QIIME2, followed by construction of a phylogenetic tree. The tree was then visualized using the R package ggtree. LEfSe analysis was conducted to identify significant correlations between bacterial taxa and different groups (Segata et al., 2011). In this study, LDA (Linear discriminant analysis) > 4 was used as the criterion for LEfSe. Microbial networks were visualized based on Gephi (0.10). *Zi-Pi* was calculated and visualized based on ggClusterNet (0.1.0) and ggplot2 (3.4.4) package of R, respectively. The roles of the nodes generally fell into four categories, as previously defined (Zhao et al., 2021): (i) peripheral nodes (*Zi* ≤ 2.5 and *Pi* ≤ 0.62); (ii) connectors (*Zi* ≤ 2.5 and *Pi* > 0.62); (iii) module hubs (*Zi* > 2.5 and *Pi* ≤ 0.62); and (iv) network hubs (*Zi* > 2.5 and *Pi* > 0.62). PICRUSt2 analysis was conducted to predict functional changes in the microbiota among groups (Douglas et al., 2020). The relative abundances between the different groups were compared using Wilcoxon and multiple test corrections (Benjamini-Hochberg FDR).

## 3 Results

### 3.1 Sequencing depth and ASV composition analysis

**Figure 1:**
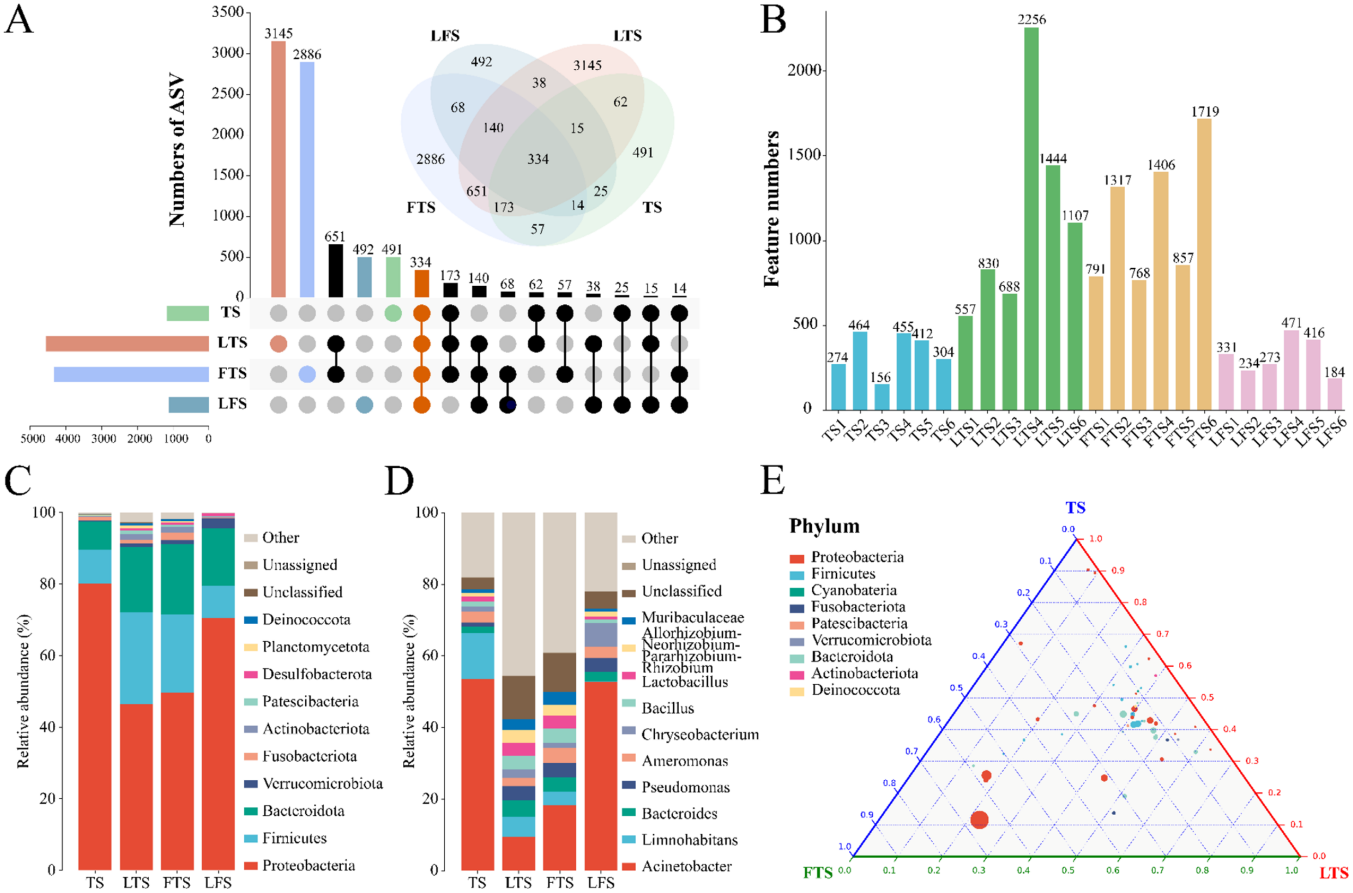
(A) Venn and upset diagrams show the shared and unique ASVs of skin microbiota among *Q. spinosa*. (B) Statistics for the number of ASVs in each sample. Relative abundances of skin microbiota at phylum (C) and genus (D) among *Q. spinosa*. (E) Analysis of skin microorganisms in aquatic life stage tadpoles (TS, LTS and FTS groups) using Ternary plot at the phylum level.

A total of 1,844,167 high-quality sequences were obtained from 24 skin samples, of which the average length of the sequences was 225 bp (Supplementary Table S1). These sequences were then clustered into ASVs, and 8,593 ASVs were obtained. ASVs among skin samples ranged from 156 (TS3) to 2,256 (LTS4), with a mean of 416 (Figure 1B). The rarefaction curves, Shannon index curves and species accumulation curves were constructed based on ASVs and showed that the depth of the sequencing results was sufficient (Supplementary Figures S1A,B,D). The milder status of the rank abundance curves in the LTS and FTS groups suggested a more uniform distribution of bacteria (Supplementary Figure S1C). In this study, 491 ASVs were unique to tadpoles, 3,145 ASVs were specific to tadpoles during hindlimb development, 2,886 ASVs were specific to tadpoles during forelimb development, 492 ASVs were specific to land frogs, and 334 ASVs were common to all groups (Figure 1A). Notably, the fewest unique ASVs were observed in the TS and LFS group for microbiota samples.

### 3.2 Microbial diversity analysis

**Figure 2:**
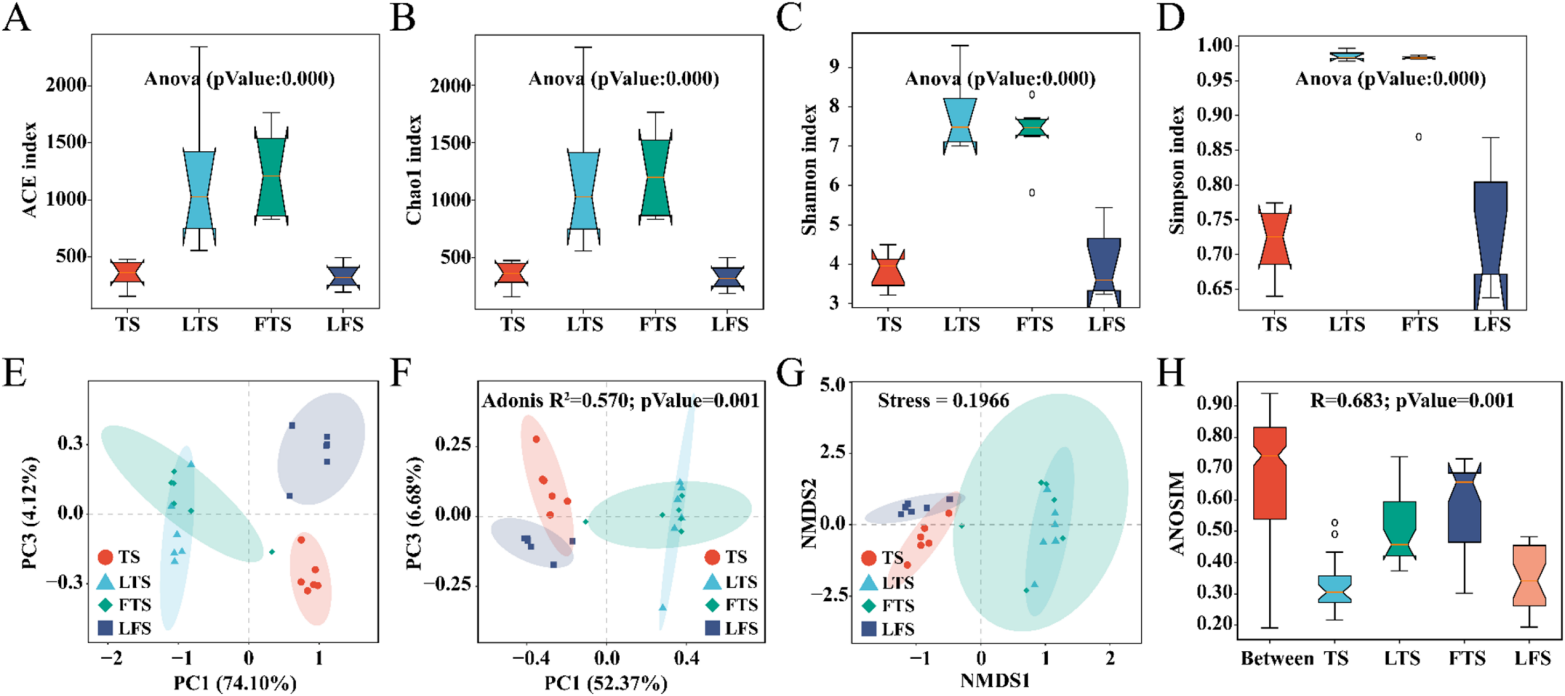
(A–D) Comparison of alpha-diversity in the skin microbiota among different *Q. spinosa* development stages. (E–H) One-way ANOVA and Waller-Duncan test were used to compare the differences among groups. Comparison of Beta-diversity in the skin microbiota among different *Q. spinosa* development stages. All Beta-diversity analysis based on Bray-Curtis distances reflected the degree of variation in skin microbiota among groups. Stress was used to assess the reasonableness and credibility of NMDS (G) analysis. ANOSIM (H) and PCoA (F) based on Bray-Curtis distances were conducted to evaluate the dissimilarity of NMDS analysis with adjusted *p* < 0.05, which was considered significant.

Overall, the coverage of skin microbiota was higher than 99.7%, indicating the reliability of the sequencing and data processing results. The ACE, Chao1, Shannon, and Simpson indexes of the skin microbiota in the LTS and FTS group were dramatically higher than those in the TS and LFS groups (Figures 2A–D; Supplementary Tables S2, S5). However, there was no significant difference between TS with LFS and LTS with FTS. Significant separation in skin microbiota was observed in all comparison sets except between the LTS and FTS groups (Figures 2E–H).

### 3.3 Microbial abundance and composition

The dominant phyla (>1%) common to all groups of skin microbiota were Proteobacteria (TS: 80.20%, LTS: 46.35%, FTS: 49.66%, LFS: 70.43%), Firmicutes (TS: 9.45%, LTS: 25.84%, FTS: 21.88%, LFS: 9.03%), and Bacteroidota (TS: 7.83%, LTS: 18.17%, FTS: 19.61%, LFS: 16.15%) (Figure 1C). Verrucomicrobiota was the dominant phylum shared by the LTS, FTS and LFS groups, and Fusobacteriota was the dominant phylum shared by the TS and FTS groups (Figure 1C). Whereas Actinobacteriota was the dominant phylum specific to the LTS and FTS groups (Figure 1C). The dominant genera (>5%) common to all groups of skin microbiota were *Acinetobacter* (TS: 53.60%, LTS: 9.38%, FTS: 18.30%, LFS: 52.80%) and *Limnohabitans* (TS: 12.83%, LTS: 5.72%, FTS: 3.81%, RD: 0.03%) (Figure 1D). *Chryseobacterium* (6.61%) was the dominant genus specific to the LFS group (Figure 1D). The ternary plot illustrated the transition of skin microbial taxa from predominantly Proteobacteria to a diverse range of microorganisms across multiple clades as the aquatic phase progressed (Figure 1E). This indicates that the skin microbial community undergoes a shift towards a more stable trend as development progresses.

**Figure 3:**
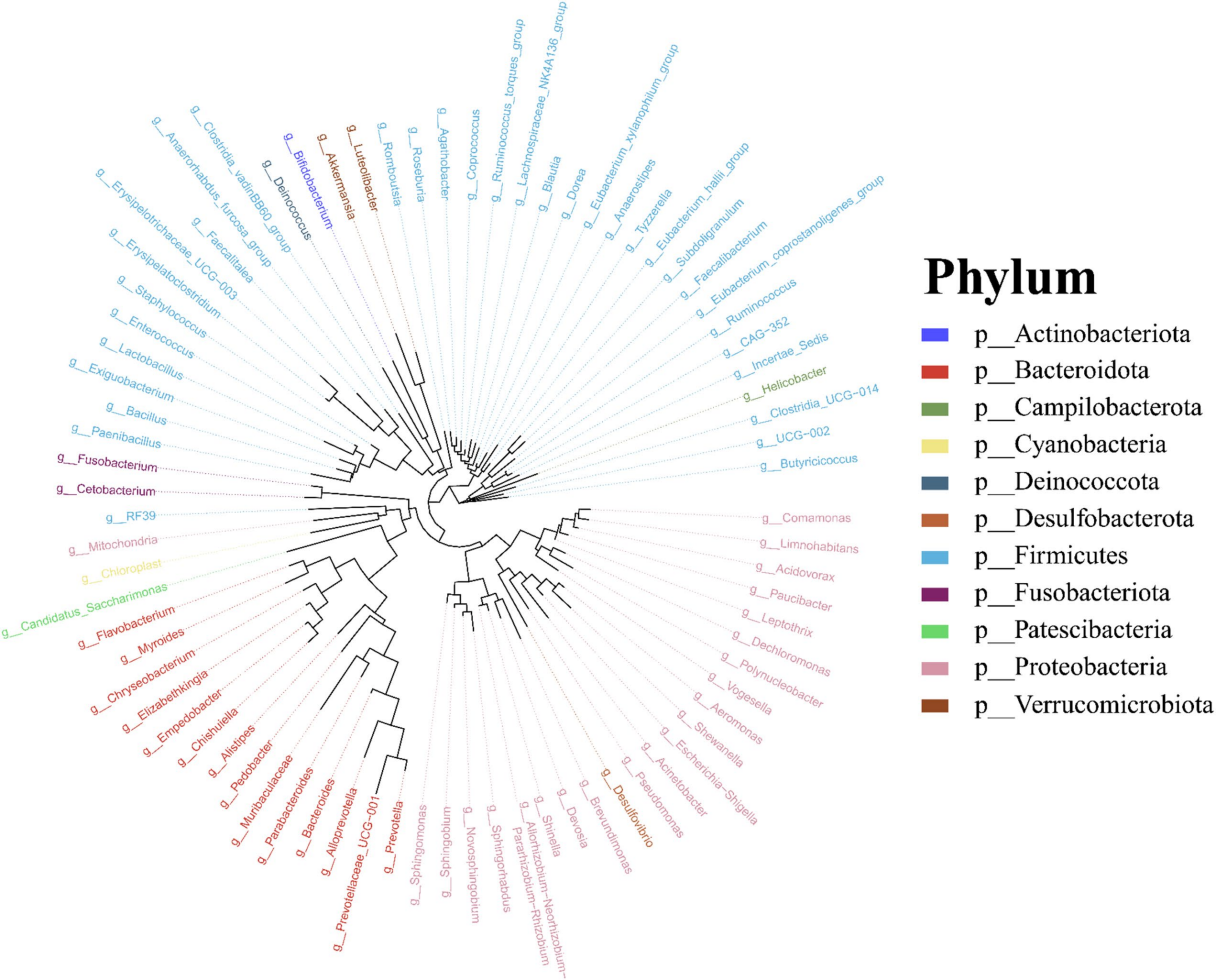
The phylogenetic tree of skin microbiota in *Q. spinosa* during metamorphosis.

Molecular phylogenetic analysis of bacteria can elucidate the evolutionary processes of the species in question, shedding light on the history and mechanisms of bacterial evolution. Representative sequences with the highest abundance of features at the genus taxonomic level were chosen, and a phylogenetic evolutionary tree was generated using Fast Tree software based on the maximum likelihood method. The resulting cyclorama visually presented the phylogenetic evolutionary relationships of skin bacteria in *Q. spinosa* during the developmental stages (Figure 3). For all periods, Proteobacteria, Firmicutes and Bacteroidota still predominate as the main three taxa in the evolution of skin microorganisms (Figure 3).

### 3.4 Microbiota variation and indicator taxa

**Figure 4:**
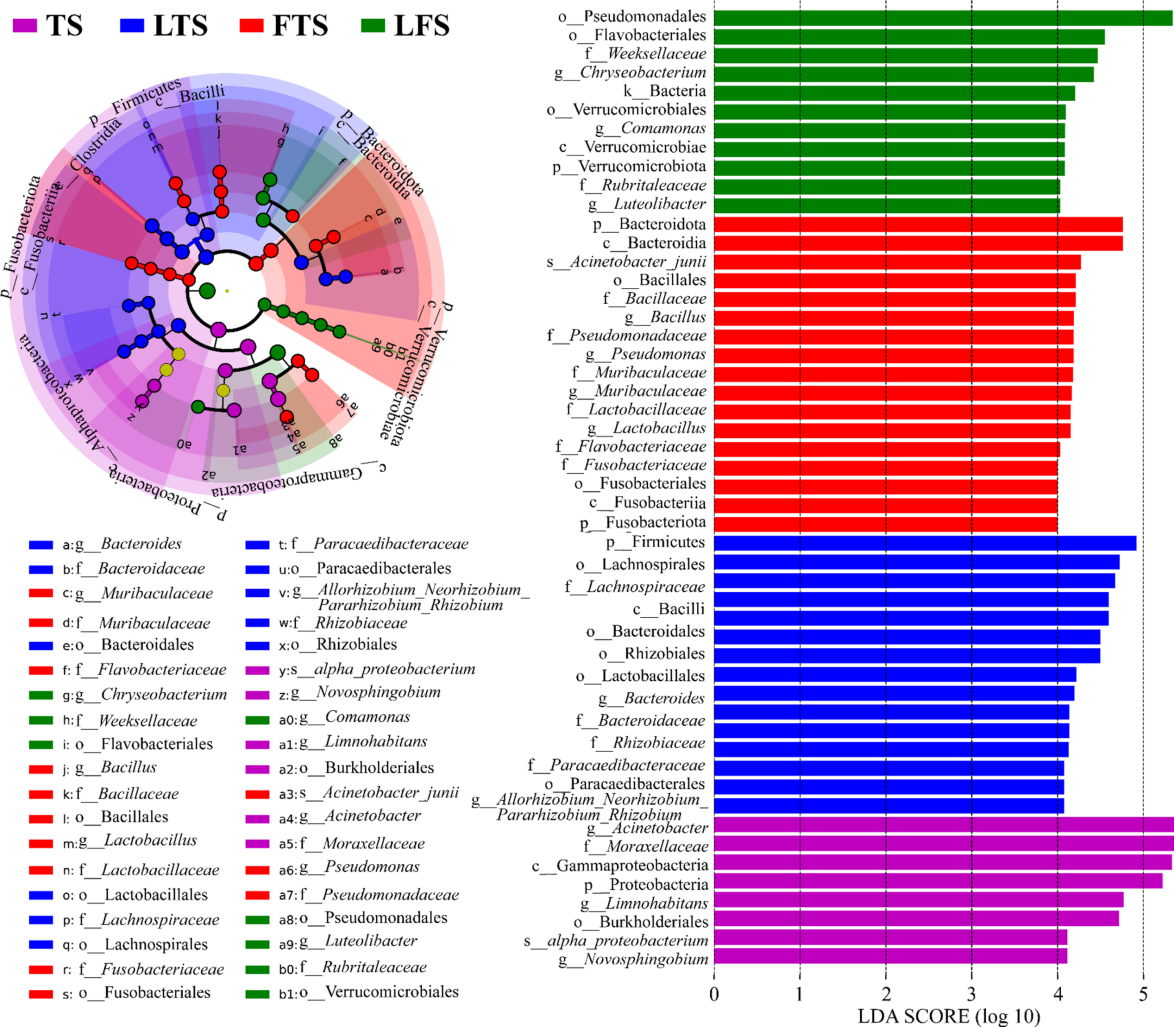
Differences in skin microbiota of *Q. spinosa* among developmental stages. The results were derived using LEfSe analysis with *LDA* > 4 and *p* < 0.05 considered significant.

The four-step rank sum test for skin microbial phylum level revealed a total of 23 phylum abundances that were significantly different (*p* < 0.05), and 17 phyla were significantly different after *P*-correction (Supplementary Table S4). LEfSe analysis revealed that Proteobacteria was significantly enriched in tadpoles’ skin, Firmicutes was significantly enriched in hindlimb tadpoles’ skin, Bacteroidota and Fusobacteriota were abundant in forelimb tadpoles’ skin, and Verrucomicrobiota was significantly enriched in land frog’s skin (Figure 4). At the genus level, *Acinetobacter*, *Novosphingobium* and *Limnohabitans* were enriched in the TS group, *Bacteroides* and *Allorhizobium_Neorhizobium_Pararhizobium_Rhizobium* were enriched in the LTS group, *Lactobacillus*, *Bacillus*, *Muribaculaceae* and *Pseudomonas* were enriched in the FTS group, and *Luteolibacter*, *Comamonas* and *Chryseobacterium* were enriched in the LFS group (Figure 4). Notably, certain microbial species, such as *alpha_proteobacterium* in TS group and *Acinetobacter_junii* in FTS group, were significantly enriched in the skin (Figure 4).

**Figure 5:**
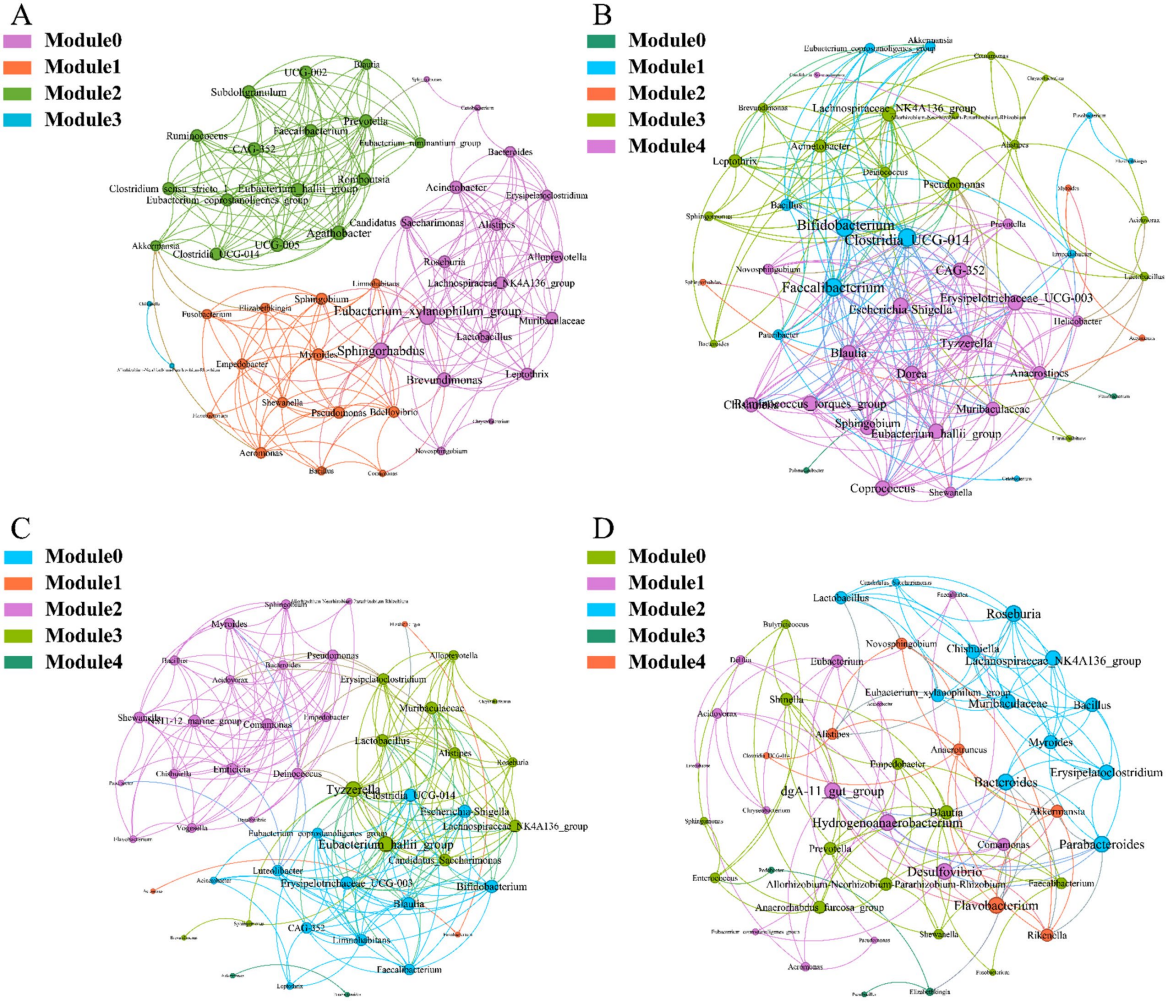
Co-occurrence network of *Q. spinosa* during metamorphosis. (A) TS group; (B) LTS group; (C) FTS group; (D) LFS group.

To investigate the interactions between microorganisms at different developmental stages, ecological networks were constructed for each of the four stages of microbial communities. Significant differences in microbial co-occurrence relationships on the skin surface at different stages are evident (Figure 5). Based on the negative correlation connection and clustering coefficient, the stability of microbial communities in each group was ranked as LTS, FTS, LFS, TS (Table 3). The results indicated that the skin microorganisms underwent notable changes throughout the development process. The microbial community showed a progression from a low-equilibrium state to a high-equilibrium state, followed by a return to a low-equilibrium state after habitat alterations.

**Table 3:** Topological characteristics of networks.

Network properties	TS	LTS	FTS	LFS
Number of nodes	49	49	48	48
Number of edges	269	241	229	142
Modularity	0.575	0.313	0.464	0.558
Number of communities	4	5	5	5
Network diameter	6	7	8	10
Network density	0.229	0.205	0.203	0.126
Average shortest path length	2.795	2.507	2.698	4.308
Average clustering coefficient	0.75	0.515	0.631	0.542
Negative	22.68%	43.15%	37.99%	23.24%
Positive	77.32%	56.85%	62.01%	76.76%

The possible topological roles of nodes in the skin were determined by their within-module connectivity (*Zi*) and among-module connectivity (*Pi*). Most nodes were classified as peripherals, with most of their connections made within their own modules. Four nodes were identified as connectors, being highly connected to several modules. Three connecters belonged to LFS group and were affiliated with *Bacteroides* (0.0272), *Parabacteroides* (0.0094) and *Flavobacterium* (0.0158) (Supplementary Table S3). *Pseudomonas* (0.0385) belonged to LTS group (Supplementary Table S3).

### 3.5 Functional prediction of microbiota

**Figure 6:**
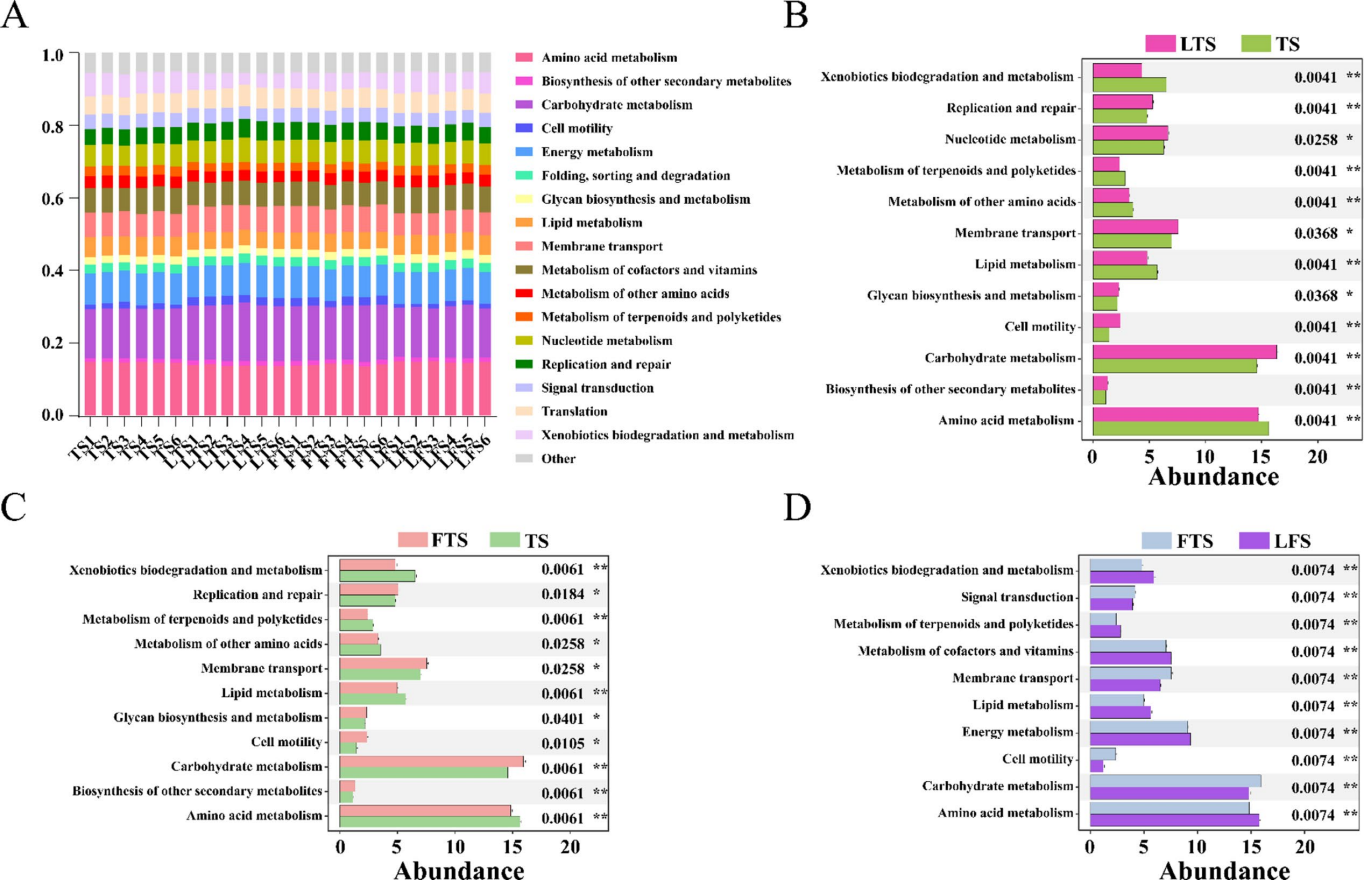
Relative abundances of skin ASVs assigned to level-2 KEGG pathways of *Q. spinosa*. PICRUSt2 was conducted to determine the predicted function. Wilcoxon and multiple test correction (Benjamini-Hochberg FDR) were used to compare the differences among groups. Data are presented as mean ± SD. *p < 0.05, **p < 0.01, ***p < 0.001.

PICRUSt2 analysis revealed 17 dominant KEGG leve_2 pathways (>1%) associated with the skin microbiota and remarkable divergence was mainly identified in the metabolism-related KEGG pathway (Figure 6A). A total of seven pathways were significantly enriched in three comparison sets, including amino acid metabolism, carbohydrate metabolism, cell motility, lipid metabolism, membrane transport, metabolism of terpenoids and polyketides and xenobiotics biodegradation and metabolism (Figures 6B–D). The functional pathways in the LTS and FTS groups showed great similarity with no significant differences between the two groups.

## 4 Discussion

### 4.1 Impact of develop on microbiota diversity

The alpha diversity of microbiota serves as an indicator of species abundance and diversity, commonly used to assess animal health (Longo and Zamudio, 2017). In ecological perception, higher diversity can be considered more resilient ecosystems (Schommer and Gallo, 2013). Changes in an animal’s health often correlate with alterations in the alpha diversity of microbiota (Jiang et al., 2022). In addition, changes in the alpha diversity of the skin microbiota often occur during development (Fieschi-Meric et al., 2023; Tong et al., 2020). This study revealed that the alpha diversity of skin microbiota in tadpoles and landing frogs was significantly lower than hindlimb tadpoles and forelimb tadpoles, with no significant differences observed between tadpoles and shore frogs, and between hind-limbed tadpoles and fore-limbed tadpoles, respectively. Furthermore, the alpha diversity of skin microbiota in tadpole stages (TS, LTS, and FTS groups) showed a significant increase with developmental maturity, a trend supported by findings in amphibians like *Hynobius maoershanensis* (Ning, 2022) and *Rana cascadae* (Kueneman et al., 2014). Similarly, the co-occurrence network results showed higher ecological stability of microflora in the LTS and FTS groups, and significantly lower in the LFS group. However, the skin surface microorganisms are undergoing adaptive changes due to habitat variations and increased complexity in skin structural functions, potentially leading to a notable reduction in alpha diversity. Conversely, the diversity of skin microorganisms was higher in *Lithobates vibicarius* (Jimenez et al., 2019) tadpoles during development compared to juvenile and adult frogs, with no significant diversity differences between juvenile and adult frogs. These distinctions may be attributed to genetic variations among organisms and the diverse environmental requirements they must adapt to.

Similar outcomes have been observed in the microbial alpha diversity changes among other amphibian species. The skin microbial communities of *Salamandra* differ significantly before and after metamorphosis, and the host itself consistently influences these skin microbial communities (Sanchez et al., 2017). The phenomenon of gradually increasing microbial diversity during metamorphosis aligns with that seen in *Salamanders* (Hartmann et al., 2023). Additionally, a decrease in skin microbiota after the completion of metamorphosis has been observed in *Anaxyrus boreas*, demonstrating a consistent pattern (Kimball, 2016). After comprehensive analysis, the primary reasons for the changes in diversity can be summarized as follows. Firstly, there is a significant change in the habitat of amphibians from aquatic to terrestrial environments during metamorphosis. Secondly, during this developmental period, there are proven to be significant alterations in the skin tissue structure of these organisms (Kinoshita et al., 1986). As development progresses, the number of glands in the skin increases significantly (Fox, 1987; Rosenberg and Warburg, 1978), and the phagocytes and fibroblasts in the skin exhibit higher activity (Takahama et al., 1992). Furthermore, there are substantial changes in the immune system during amphibian development (Pollet, 2010). Studies have also demonstrated that the immune system of amphibian tadpoles differs from that of adults (Rollins-Smith, 1998), with tadpoles only being able to reject a limited number of transplanted antigens (Pasquier et al., 2000). Following the completion of metamorphosis, the number of lymphocytes in young frogs decreases by approximately 40% (Pasquier et al., 2000), but ultimately, the transformation from lymphoid tissue to lymphoid organs is completed (Manning and Horton, 1969). The antimicrobial peptides secreted by the skin of *Litoria ewingii* gradually increase in antibacterial activity as they develop, and their composition tends to diversify (Schadich et al., 2009).

Beta diversity statistics under the Bray-Curtis distance are based on shared species for comparison and are sensitive to changes in taxonomic abundance (Ricotta and Pavoine, 2022). The findings of this research demonstrate significant differentiation in skin microbiota across various developmental stages, but the LTS and FTS groups showing high similarity. It indicates that skin microbes evolve with the development of the organisms. Despite disturbances faced by amphibian skin microorganisms from factors like the environment, pathogenic microorganisms, and ecological imbalances (Lopez et al., 2017; Tong et al., 2023), it is the interplay between the host’s immune system and symbiotic flora that safeguards the host’s health (Hou et al., 2022). The symbiotic flora aids in repelling external pathogenic bacteria, while the immune system ensures the survival dominance of the symbiotic flora (Lopez et al., 2017; Woodhams et al., 2020). The early developmental stage of *Q. spinosa* is a critical period for the development and refinement of various physiological functions of the body (such as digestion, immunity, respiration, and blood circulation) (Hou et al., 2022; Xu et al., 2024a). This period is when microorganisms and hosts establish symbiotic relationships, but it is also vulnerable to bacterial imbalances and invasion by pathogenic bacteria, particularly during the critical landing period (LFS) (Long et al., 2020). This study underscores the significance of comprehending the evolution of skin commensal flora throughout development to inform healthy farming practices, health evaluations, and amphibian conservation efforts.

### 4.2 Composition of skin microbes and indicator taxa

Similar to other amphibians (Hou et al., 2022; Jiang et al., 2022; Karlsen et al., 2017; Long et al., 2020; Zhu et al., 2024), Proteobacteria, Firmicutes, and Bacteroidota are the predominant microbiota of the skin, albeit with varying abundance across different phyla. Proteobacteria harbor many opportunistic pathogens, while most bacterial genera in Firmicutes are considered beneficial, helping maintain the acidic environment of the host skin and producing active substances through fermentation to resist pathogenic microorganisms (Huang et al., 2020; Xu et al., 2024a,b). Bacteroidota members are closely associated with the host’s immune and metabolic processes (Federici et al., 2015). This study found that the abundance of Proteobacteria significantly increased with development in *Q. spinosa*, indicating heightened metabolism and immunity in the early stages. The abundance of Proteobacteria among skin microorganisms decreased notably during the tadpole stage (TS, LTS and FTS group) and increased significantly post-landing (LFS group), while the abundance of Firmicutes exhibited the opposite trend. Similar patterns were observed in the development of other amphibians (Fieschi-Meric et al., 2023). We hypothesize that continuous adaptation to the aqueous environment leads to the colonization of beneficial symbiotic microorganisms on the skin. However, changes in habitat and skin functions alter the original ecology, resulting in a re-selection of host-microorganism interactions.

Interestingly, our results show that *Acinetobacter* sp. increased rapidly between the forelimb tadpoles and the landing frogs. In cases of disease, *Acinetobacter* sp. is typically associated with skin infections, ulcers, and necrosis, among other symptoms (Zhu et al., 2024). It is also commonly one of the primary causative agents of pneumonia, meningitis, and septicemia in frogs (Lee et al., 2017). In contrast, *Acinetobacter* sp. have a significant inhibitory effect on the pathogenic fungi of amphibian “chytridiomycosis” (Fieschi-Meric et al., 2023), and also support and protect microbial communities in biological systems, which are important for biofilm development (Zhu et al., 2024). We speculate that the enrichment of *Acinetobacter* sp. in the TS group may be for pre-skin development and colonization of symbiotic microbial communities. Conversely, the enrichment in the LFS group may be to resist colonization by *Batrachochytrium dendrobatidis* and to reorganize the microbial community. Thus, the developmental window between forelimb tadpoles and land frog could be key in establishing host defences against pathogens.

LEfSe analysis revealed several genera with significant differences in abundance among groups, including *Acinetobacter*, *Novosphingobium*, *Limnohabitans*, *Bacteroides*, *Bacillus*, *Lactobacillus*, *Muribaculaceae*, *Pseudomonas*, *Luteolibacter*, *Comamonas*, *Chryseobacterium* and *Allorhizobium_Neorhizobium_Pararhizobium_Rhizobium*. In aquaculture production, *Bacillus*, *Bacteroides* and *Lactobacillus* were commonly used as probiotics to maintain the health of farmed animals (Liu et al., 2023; Xu et al., 2024a; Zhu et al., 2024). For instance, *B. subtilis* can inhibit the growth of *Vibro alginolyticus* and *A. bouvetii* (Qi et al., 2020; Song et al., 2009). *B. velezensis* has an antagonistic effect on the pathogenic bacteria of aquatic conditions, including *Edwardsiella tarda*, *A. veronii* and *A. hydrophila* (Gao, 2019). The feed adding *B. amyloliquefaciens* and *B. subtilis* also have the role of enhancing the immunity of breeding objects and promoting growth (Lai et al., 2020; Lu et al., 2021). *Bacteroides* can inhibit harmful bacteria, provide more active protease and lipase, and promote feed utilization (Christensen and Bruggemann, 2014). *Lactobacillus* isolated from bullfrogs have been found to regulate host immune function and combat pathogens (Pereira et al., 2018). The co-occurrence network analysis revealed variations in skin microbial ecology at different stages, influencing the evaluation of host status through the identification of beneficial bacteria indicated by LEfSe. This information can be valuable for guiding aquaculture and preventive measures.

Existing reports indicate that bacterial diseases in the Chinses spiny frog often result from the synergistic action of multiple pathogenic bacteria, manifesting as a single disease with multiple symptoms or multiple diseases with a single symptom (Jiang, 2022). Based on outbreak causes and prevention and control experiences in breeding farms, the following points should be taken into consideration for the prevention and control of opportunistic pathogens during development: (1) Strict epidemic prevention measures should be implemented for the entry and exit of breeding personnel and animals into and out of the breeding area, even extending to the specific breeding ponds to prevent cross-infection of microorganisms. At the same time, mixing of breeding animals from different ponds should be prohibited. (2) Regular microbial prevention and control measures should be taken during the metamorphosis stages (LTS, FTS, and LFS), including the breeding area, related facilities and equipment, and regular incorporation of beneficial bacteria into feed. (3) Individuals or groups showing signs of illness should be promptly isolated, and the isolation ponds should be strictly disinfected.

### 4.3 Functions during microbial development

Symbiotic microorganisms residing on the host skin are vital components that play a crucial role in defending against pathogen invasion, regulating pH levels, influencing metabolism, and supporting immune system development (Abarca et al., 2018; Harris-Tryon and Grice, 2022; Pereira et al., 2018). In this study, PICRUSt was utilized to evaluate the predicted functions of the skin microbiota. Variations in skin microbial function are observed over develop, with a particular emphasis on amino acid metabolism, carbohydrate metabolism, cell motility, lipid metabolism, membrane transport, metabolism of terpenoids and polyketides, and xenobiotics biodegradation and metabolism. The results indicate that skin microbes play a crucial role in metabolic functions, while the enrichment of cell motility and membrane transport pathways suggests that the skin symbiotic flora also aids in the developmental refinement of the host’s skin.

## 5 Conclusion

In this study, we systematically examined the skin microorganisms present during metamorphosis in *Q. spinosa*, Echinococcus auratus. Our findings demonstrate that the composition and diversity of skin microorganisms undergo changes in response to developmental and environmental factors. The dominant phyla of skin microorganisms identified were Proteobacteria, Firmicutes and Bacteroidota, with their abundance being influenced by the host’s developmental stage. Key bacterial indicators highlighted by LEfSe included *Bacteroides*, *Bacillus*, *Lactobacillus*, and *Acinetobacter*. Moreover, the functions performed by skin microbes varied significantly during development, primarily focusing on metabolic activities. The comprehensive analysis suggests that the transition from forelimb tadpole to frog landing poses a high risk of pathogenic microorganism invasion, making it a critical period for successful metamorphosis in *Q. spinosa*. This study underscores the importance of host skin microbes in amphibian development and habitat, offering potential implications for managing amphibian metamorphosis in relation to microbial communities.

## Data Availability

The merged sequences are deposited in the NCBI SRA database under accession number PRJNA1058082.
