# Supplementary material for: Skin microbiota during metamorphosis of Quasipaa spinosa: guidance for maintaining mucosal symbiotic microbial flora homeostasis in early life of frogs
Source: Front Microbiol. 2024 Nov 14;15:1453617. doi: 10.3389/fmicb.2024.1453617 (PMC11602500; doi:10.3389/fmicb.2024.1453617)

| **Table S1** Statistical table of the sample sequencing data | | | | | | |
| --- | --- | --- | --- | --- | --- | --- |
| Sample | Raw Reads number (PE) | Clean Reads number (PE) | Total length (bp) | Average length (bp) | Q 20 | Q 30 |
| TS1 | 82,342 | 52,070 | 23,499,432 | 225 | 96.73 | 94.41 |
| TS2 | 86,865 | 55,784 | 25,173,486 | 225 | 96.83 | 94.59 |
| TS3 | 86,011 | 54,239 | 24,478,852 | 225 | 96.65 | 94.33 |
| TS4 | 76,728 | 47,290 | 21,340,484 | 225 | 96.77 | 94.53 |
| TS5 | 83,852 | 54,892 | 24,770,630 | 225 | 96.83 | 94.58 |
| TS6 | 106,133 | 40,503 | 18,267,311 | 225 | 95.5 | 92.68 |
| LTS1 | 62,964 | 38,710 | 17,466,321 | 225 | 95.76 | 92.98 |
| LTS2 | 99,178 | 63,965 | 28,865,645 | 225 | 96.47 | 94.12 |
| LTS3 | 145,612 | 77,295 | 34,876,838 | 225 | 96.69 | 94.31 |
| LTS4 | 157,984 | 85,498 | 38,571,126 | 225 | 96.67 | 94.23 |
| LTS5 | 134,844 | 77,101 | 34,782,850 | 225 | 96.69 | 94.29 |
| LTS6 | 170,126 | 99,309 | 44,804,716 | 225 | 96.73 | 94.42 |
| FTS1 | 140,631 | 100,292 | 45,282,987 | 225 | 97.69 | 95.97 |
| FTS2 | 183,040 | 134,854 | 60,883,632 | 225 | 97.68 | 95.9 |
| FTS3 | 128,680 | 99,070 | 44,734,978 | 225 | 97.83 | 96.22 |
| FTS4 | 133,929 | 102,634 | 46,337,438 | 225 | 97.63 | 95.82 |
| FTS5 | 148,335 | 100,196 | 45,236,095 | 225 | 97.66 | 95.90 |
| FTS6 | 150,499 | 116,311 | 52,506,874 | 225 | 97.75 | 96.00 |
| LFS1 | 99,691 | 80,989 | 36,573,198 | 225 | 97.84 | 96.31 |
| LFS2 | 95,616 | 68,951 | 31,135,734 | 225 | 97.86 | 96.34 |
| LFS3 | 93,410 | 75,960 | 34,301,655 | 225 | 97.89 | 96.38 |
| LFS4 | 102,484 | 71,675 | 32,354,280 | 225 | 96.77 | 94.64 |
| LFS5 | 116,700 | 82,625 | 37,292,833 | 225 | 96.75 | 94.61 |
| LFS6 | 86,114 | 63,954 | 28,871,230 | 225 | 96.60 | 94.38 |
| Sum | 2,771,768 | 1,844,167 | - | - | - | - |

Note: Q20 means the ratio of bases with a quality value of 20 (99% accuracy) to total bases; Q30 means the ratio of bases with a quality value of 30 (99.9% accuracy) to total bases.

| Table S2 Significance analysis for alpha diversity | | | | | |
| --- | --- | --- | --- | --- | --- |
| Groups | P-Value | | | | Method (Anova) |
|  | ACE | Chao1 | Shannon | Simpson |  |
| TS vs LTS | 0.011 | 0.011 | 0.000 | 0.000 | t-test |
| TS vs FTS | 0.001 | 0.001 | 0.000 | 0.000 | t-test |
| TS vs LFS | 0.814 | 0.815 | 0.711 | 0.809 | t-test |
| LTS vs FTS | 0.904 | 0.904 | 0.373 | 0.299 | t-test |
| LTS vs LFS | 0.010 | 0.010 | 0.000 | 0.000 | t-test |
| FTS vs LFS | 0.000 | 0.000 | 0.000 | 0.000 | t-test |

| Table S3 Co-occurrence network characteristics | | | | | | | | | | |
| --- | --- | --- | --- | --- | --- | --- | --- | --- | --- | --- |
| Genus | Abundance | Module Class | Degree | Closeness Centrality | Betweenness Centrality | Custering Coefficient | *Zi* | *Pi* | Node Type | Group |
| *Comamonas* | 0.0042 | Module1 | 4 | 0.37 | 0.01 | 0.83 | -1.7937 | 0.375 | Peripherals | TS |
| *Romboutsia* | 0.0022 | Module2 | 14 | 0.29 | 0 | 0.91 | 0.4714 | 0.0000 | Peripherals | TS |
| *Acinetobacter* | 0.5360 | Module0 | 14 | 0.38 | 0.09 | 0.73 | 0.8480 | 0.0000 | Peripherals | TS |
| *Leptothrix* | 0.0095 | Module0 | 13 | 0.34 | 0.02 | 0.76 | 0.6095 | 0.0000 | Peripherals | TS |
| *Prevotella* | 0.0060 | Module2 | 14 | 0.29 | 0 | 0.91 | 0.4714 | 0.0000 | Peripherals | TS |
| *Sphingobium* | 0.0032 | Module1 | 12 | 0.41 | 0.04 | 0.65 | 0.9728 | 0.2778 | Peripherals | TS |
| *Cetobacterium* | 0.0021 | Module0 | 3 | 0.35 | 0.14 | 0.33 | -1.7755 | 0.0000 | Peripherals | TS |
| *Limnohabitans* | 0.1283 | Module1 | 8 | 0.4 | 0.03 | 0.68 | -1.0032 | 0.4688 | Peripherals | TS |
| *Lactobacillus* | 0.0142 | Module0 | 13 | 0.34 | 0 | 0.91 | 0.6095 | 0.0000 | Peripherals | TS |
| *Bdellovibrio* | 0.0014 | Module1 | 11 | 0.41 | 0.02 | 0.62 | 0.1824 | 0.3967 | Peripherals | TS |
| *Clostridia_UCG-014* | 0.0009 | Module2 | 13 | 0.33 | 0.01 | 0.92 | 0.0000 | 0.0000 | Peripherals | TS |
| *Faecalibacterium* | 0.0065 | Module2 | 14 | 0.29 | 0 | 0.91 | 0.4714 | 0.0000 | Peripherals | TS |
| *Elizabethkingia* | 0.0108 | Module1 | 9 | 0.41 | 0.07 | 0.75 | 0.1824 | 0.1975 | Peripherals | TS |
| *Shewanella* | 0.0035 | Module1 | 10 | 0.39 | 0.01 | 0.78 | 0.5776 | 0.1800 | Peripherals | TS |
| *Brevundimonas* | 0.0044 | Module0 | 16 | 0.38 | 0.05 | 0.55 | 0.3710 | 0.3750 | Peripherals | TS |
| *Chishuiella* | 0.0016 | Module3 | 1 | 0.02 | 0 | 0 | 0.0000 | 0.0000 | Peripherals | TS |
| *Myroides* | 0.0016 | Module1 | 12 | 0.41 | 0.04 | 0.65 | 0.9728 | 0.2778 | Peripherals | TS |
| *Allorhizobium-Neorhizobium-Pararhizobium-Rhizobium* | 0.0105 | Module3 | 1 | 0.02 | 0 | 0 | 0.0000 | 0.0000 | Peripherals | TS |
| *Clostridium_sensu_stricto_1* | 0.0014 | Module2 | 13 | 0.33 | 0.01 | 0.92 | 0.0000 | 0.0000 | Peripherals | TS |
| *Alloprevotella* | 0.0012 | Module0 | 13 | 0.34 | 0 | 0.91 | 0.6095 | 0.0000 | Peripherals | TS |
| *Empedobacter* | 0.0051 | Module1 | 10 | 0.39 | 0.01 | 0.78 | 0.5776 | 0.1800 | Peripherals | TS |
| *Eubacterium_hallii_group* | 0.0008 | Module2 | 15 | 0.33 | 0.02 | 0.85 | 0.9428 | 0.0000 | Peripherals | TS |
| *Subdoligranulum* | 0.0035 | Module2 | 14 | 0.29 | 0 | 0.91 | 0.4714 | 0.0000 | Peripherals | TS |
| *UCG-005* | 0.0009 | Module2 | 15 | 0.33 | 0.02 | 0.85 | 0.9428 | 0.0000 | Peripherals | TS |
| *Chryseobacterium* | 0.0141 | Module0 | 2 | 0.25 | 0 | 1 | -2.0140 | 0.0000 | Peripherals | TS |
| *CAG-352* | 0.0011 | Module2 | 14 | 0.29 | 0 | 0.91 | 0.4714 | 0.0000 | Peripherals | TS |
| *Candidatus_Saccharimonas* | 0.0020 | Module0 | 13 | 0.34 | 0 | 0.91 | 0.6095 | 0.0000 | Peripherals | TS |
| *Aeromonas* | 0.0303 | Module1 | 11 | 0.42 | 0.12 | 0.6 | 0.9728 | 0.1653 | Peripherals | TS |
| *Bacteroides* | 0.0176 | Module0 | 11 | 0.36 | 0.05 | 0.84 | 0.1325 | 0.0000 | Peripherals | TS |
| *Roseburia* | 0.0024 | Module0 | 13 | 0.34 | 0 | 0.91 | 0.6095 | 0.0000 | Peripherals | TS |
| *Pseudomonas* | 0.0109 | Module1 | 12 | 0.41 | 0.04 | 0.65 | 0.9728 | 0.2778 | Peripherals | TS |
| *Alistipes* | 0.0012 | Module0 | 13 | 0.34 | 0 | 0.91 | 0.6095 | 0.0000 | Peripherals | TS |
| *Fusobacterium* | 0.0081 | Module1 | 9 | 0.41 | 0.07 | 0.75 | 0.1824 | 0.1975 | Peripherals | TS |
| *Erysipelatoclostridium* | 0.0009 | Module0 | 10 | 0.32 | 0 | 1 | -0.1060 | 0.0000 | Peripherals | TS |
| *Eubacterium_coprostanoligenes_group* | 0.0030 | Module2 | 13 | 0.33 | 0.01 | 0.92 | 0.0000 | 0.0000 | Peripherals | TS |
| *Lachnospiraceae_NK4A136_group* | 0.0066 | Module0 | 13 | 0.34 | 0 | 0.91 | 0.6095 | 0.0000 | Peripherals | TS |
| *Agathobacter* | 0.0015 | Module2 | 16 | 0.37 | 0.17 | 0.74 | 0.9428 | 0.1172 | Peripherals | TS |
| *Sphingomonas* | 0.0008 | Module0 | 2 | 0.34 | 0.13 | 0 | -2.2525 | 0.5000 | Peripherals | TS |
| *Bacillus* | 0.0142 | Module1 | 7 | 0.38 | 0.01 | 0.67 | -1.0032 | 0.4082 | Peripherals | TS |
| *UCG-002* | 0.0031 | Module2 | 14 | 0.29 | 0 | 0.91 | 0.4714 | 0.0000 | Peripherals | TS |
| *Akkermansia* | 0.0010 | Module2 | 10 | 0.39 | 0.28 | 0.53 | -2.8284 | 0.4200 | Peripherals | TS |
| *Blautia* | 0.0014 | Module2 | 10 | 0.29 | 0 | 1 | -1.4142 | 0.0000 | Peripherals | TS |
| *Eubacterium_xylanophilum_group* | 0.0008 | Module0 | 19 | 0.39 | 0.1 | 0.53 | 0.8480 | 0.3878 | Peripherals | TS |
| *Eubacterium_ruminantium_group* | 0.0009 | Module2 | 10 | 0.29 | 0 | 1 | -1.4142 | 0.0000 | Peripherals | TS |
| *Muribaculaceae* | 0.0103 | Module0 | 13 | 0.34 | 0 | 0.91 | 0.6095 | 0.0000 | Peripherals | TS |
| *Novosphingobium* | 0.0296 | Module0 | 6 | 0.32 | 0.02 | 0.67 | -1.0600 | 0.0000 | Peripherals | TS |
| *Sphingorhabdus* | 0.0078 | Module0 | 19 | 0.38 | 0.1 | 0.45 | 0.1325 | 0.4875 | Peripherals | TS |
| *Flavobacterium* | 0.0022 | Module1 | 3 | 0.29 | 0.00 | 1.00 | -1.7937 | 0.0000 | Peripherals | TS |
| *Ruminococcus* | 0.0011 | Module2 | 13 | 0.33 | 0.01 | 0.92 | 0.0000 | 0.0000 | Peripherals | TS |
| *Paucibacter* | 0.0028 | Module1 | 9 | 0.45 | 0.06 | 0.50 | 0.7789 | 0.4938 | Peripherals | LTS |
| *Comamonas* | 0.0081 | Module3 | 5 | 0.36 | 0.01 | 0.50 | -0.0875 | 0.0000 | Peripherals | LTS |
| *Acinetobacter* | 0.0938 | Module3 | 13 | 0.44 | 0.05 | 0.50 | 1.2247 | 0.4734 | Peripherals | LTS |
| *Leptothrix* | 0.0111 | Module3 | 13 | 0.44 | 0.05 | 0.50 | 1.2247 | 0.4734 | Peripherals | LTS |
| *Sphingobium* | 0.0088 | Module4 | 16 | 0.44 | 0.01 | 0.84 | 0.5264 | 0.3047 | Peripherals | LTS |
| *Prevotella* | 0.0029 | Module4 | 9 | 0.37 | 0.00 | 0.92 | -0.8670 | 0.1975 | Peripherals | LTS |
| *Cetobacterium* | 0.0066 | Module1 | 1 | 0.31 | 0.00 | 0.00 | -1.6011 | 0.0000 | Peripherals | LTS |
| *Limnohabitans* | 0.0572 | Module3 | 2 | 0.28 | 0.04 | 0.00 | -1.3997 | 0.0000 | Peripherals | LTS |
| *Lactobacillus* | 0.0358 | Module3 | 6 | 0.40 | 0.02 | 0.53 | -0.5249 | 0.4444 | Peripherals | LTS |
| *Escherichia-Shigella* | 0.0066 | Module4 | 17 | 0.45 | 0.01 | 0.80 | 0.5264 | 0.3806 | Peripherals | LTS |
| *Clostridia_UCG-014* | 0.0050 | Module1 | 22 | 0.54 | 0.08 | 0.54 | 0.7789 | 0.595 | Peripherals | LTS |
| *Faecalibacterium* | 0.0055 | Module1 | 22 | 0.54 | 0.08 | 0.54 | 0.7789 | 0.595 | Peripherals | LTS |
| *Elizabethkingia* | 0.0069 | Module1 | 1 | 0.22 | 0.00 | 0.00 | -1.6011 | 0.0000 | Peripherals | LTS |
| *Shewanella* | 0.0128 | Module4 | 10 | 0.44 | 0.08 | 0.53 | -1.1457 | 0.4600 | Peripherals | LTS |
| *Brevundimonas* | 0.0095 | Module3 | 7 | 0.38 | 0.01 | 0.62 | 0.3499 | 0.2449 | Peripherals | LTS |
| *Chishuiella* | 0.0049 | Module4 | 16 | 0.44 | 0.01 | 0.84 | 0.5264 | 0.3047 | Peripherals | LTS |
| *Helicobacter* | 0.0029 | Module4 | 10 | 0.42 | 0.07 | 0.51 | -0.8670 | 0.3200 | Peripherals | LTS |
| *Myroides* | 0.0046 | Module2 | 2 | 0.28 | 0.02 | 0.00 | -0.7071 | 0.5000 | Peripherals | LTS |
| *Allorhizobium-Neorhizobium-*  *Pararhizobium-Rhizobium* | 0.0355 | Module3 | 3 | 0.31 | 0.00 | 1.00 | -0.9623 | 0.0000 | Peripherals | LTS |
| *Ruminococcus_torques_group* | 0.0035 | Module4 | 17 | 0.48 | 0.02 | 0.78 | 0.8051 | 0.2907 | Peripherals | LTS |
| *Tyzzerella* | 0.0037 | Module4 | 17 | 0.45 | 0.01 | 0.80 | 0.5264 | 0.3806 | Peripherals | LTS |
| *Empedobacter* | 0.0141 | Module1 | 5 | 0.38 | 0.08 | 0.50 | -0.6491 | 0.4800 | Peripherals | LTS |
| *Eubacterium_hallii_group* | 0.0092 | Module4 | 17 | 0.45 | 0.01 | 0.80 | 0.5264 | 0.3806 | Peripherals | LTS |
| *Anaerostipes* | 0.0027 | Module4 | 14 | 0.38 | 0.06 | 0.75 | 0.5264 | 0.1327 | Peripherals | LTS |
| *Erysipelotrichaceae_UCG-003* | 0.0056 | Module4 | 17 | 0.48 | 0.02 | 0.78 | 0.8051 | 0.2907 | Peripherals | LTS |
| *CAG-352* | 0.0041 | Module4 | 17 | 0.45 | 0.01 | 0.80 | 0.5264 | 0.3806 | Peripherals | LTS |
| *Candidatus_Saccharimonas* | 0.0046 | Module4 | 1 | 0.29 | 0.00 | 0.00 | -2.8177 | 0.0000 | Peripherals | LTS |
| *Chryseobacterium* | 0.0230 | Module3 | 1 | 0.22 | 0.00 | 0.00 | -1.8371 | 0.0000 | Peripherals | LTS |
| *Aeromonas* | 0.0238 | Module2 | 2 | 0.26 | 0.00 | 0.00 | 1.4142 | 0.0000 | Peripherals | LTS |
| *Bacteroides* | 0.0461 | Module3 | 4 | 0.33 | 0.00 | 0.50 | -0.5249 | 0.0000 | Peripherals | LTS |
| *Dorea* | 0.0029 | Module4 | 17 | 0.48 | 0.02 | 0.78 | 0.8051 | 0.2907 | Peripherals | LTS |
| *Pseudomonas* | 0.0385 | Module3 | 14 | 0.45 | 0.06 | 0.52 | -0.0875 | 0.6429 | Connectors | LTS |
| *Alistipes* | 0.0048 | Module3 | 7 | 0.38 | 0.02 | 0.48 | 0.7873 | 0.0000 | Peripherals | LTS |
| *Fusobacterium* | 0.0032 | Module1 | 2 | 0.28 | 0.04 | 0.00 | -1.1251 | 0.0000 | Peripherals | LTS |
| *Eubacterium_coprostanoligenes_group* | 0.0034 | Module1 | 8 | 0.42 | 0.01 | 0.82 | 0.3029 | 0.4688 | Peripherals | LTS |
| *Lachnospiraceae_NK4A136_group* | 0.0194 | Module3 | 15 | 0.50 | 0.09 | 0.44 | 1.6622 | 0.5244 | Peripherals | LTS |
| *Sphingomonas* | 0.0038 | Module3 | 7 | 0.39 | 0.01 | 0.76 | 0.7873 | 0.0000 | Peripherals | LTS |
| *Bacillus* | 0.0385 | Module1 | 11 | 0.47 | 0.07 | 0.53 | 1.2550 | 0.5289 | Peripherals | LTS |
| *Akkermansia* | 0.0041 | Module1 | 8 | 0.42 | 0.01 | 0.82 | 0.3029 | 0.4688 | Peripherals | LTS |
| *Polynucleobacter* | 0.0128 | Module0 | 1 | 0.02 | 0.00 | 0.00 | 0.0000 | 0.0000 | Peripherals | LTS |
| *Blautia* | 0.0105 | Module4 | 17 | 0.48 | 0.02 | 0.78 | 0.8051 | 0.2907 | Peripherals | LTS |
| *Muribaculaceae* | 0.0314 | Module4 | 15 | 0.51 | 0.08 | 0.54 | -1.1457 | 0.6044 | Peripherals | LTS |
| *Novosphingobium* | 0.0123 | Module4 | 9 | 0.37 | 0.00 | 0.92 | -0.8670 | 0.1975 | Peripherals | LTS |
| *Deinococcus* | 0.0053 | Module3 | 9 | 0.39 | 0.08 | 0.5 | 0.3499 | 0.4444 | Peripherals | LTS |
| *Sphingorhabdus* | 0.0180 | Module2 | 2 | 0.31 | 0.02 | 0.00 | -0.7071 | 0.5000 | Peripherals | LTS |
| *Flavobacterium* | 0.0104 | Module0 | 1 | 0.02 | 0.00 | 0.00 | 0.0000 | 0.0000 | Peripherals | LTS |
| *Coprococcus* | 0.0027 | Module4 | 17 | 0.48 | 0.02 | 0.78 | 0.8051 | 0.2907 | Peripherals | LTS |
| *Acidovorax* | 0.0032 | Module3 | 4 | 0.34 | 0.01 | 0.67 | -0.9623 | 0.3750 | Peripherals | LTS |
| *Bifidobacterium* | 0.0076 | Module1 | 22 | 0.54 | 0.08 | 0.54 | 0.7789 | 0.5950 | Peripherals | LTS |
| *Paucibacter* | 0.0039 | Module2 | 3 | 0.37 | 0.01 | 0.67 | -1.8368 | 0.4444 | Peripherals | FTS |
| *Vogesella* | 0.0033 | Module2 | 9 | 0.37 | 0.01 | 0.67 | 0.1933 | 0 | Peripherals | FTS |
| *Comamonas* | 0.0143 | Module2 | 13 | 0.38 | 0.03 | 0.64 | 1.3534 | 0 | Peripherals | FTS |
| *Acinetobacter* | 0.1830 | Module0 | 5 | 0.32 | 0 | 1 | -1.8074 | 0 | Peripherals | FTS |
| *Leptothrix* | 0.0051 | Module0 | 5 | 0.32 | 0 | 1 | -1.8074 | 0 | Peripherals | FTS |
| *Sphingobium* | 0.0082 | Module2 | 9 | 0.32 | 0 | 0.97 | 0.1933 | 0 | Peripherals | FTS |
| *Limnohabitans* | 0.0381 | Module0 | 13 | 0.44 | 0.05 | 0.73 | 1.291 | 0.2604 | Peripherals | FTS |
| *Lactobacillus* | 0.0361 | Module3 | 14 | 0.4 | 0.01 | 0.78 | 0.4466 | 0.4592 | Peripherals | FTS |
| *Escherichia-Shigella* | 0.0037 | Module0 | 15 | 0.45 | 0.03 | 0.76 | 0.2582 | 0.48 | Peripherals | FTS |
| *Clostridia_UCG-014* | 0.0036 | Module0 | 15 | 0.45 | 0.03 | 0.76 | 0.2582 | 0.48 | Peripherals | FTS |
| *Faecalibacterium* | 0.0030 | Module0 | 12 | 0.4 | 0.02 | 0.76 | 0.7746 | 0.2778 | Peripherals | FTS |
| *Elizabethkingia* | 0.0185 | Module1 | 2 | 0.27 | 0.08 | 0 | -0.7071 | 0.5 | Peripherals | FTS |
| *Shewanella* | 0.0143 | Module2 | 12 | 0.33 | 0.01 | 0.64 | 1.0634 | 0 | Peripherals | FTS |
| *Brevundimonas* | 0.0060 | Module3 | 1 | 0.25 | 0 | 0 | -1.9979 | 0 | Peripherals | FTS |
| *Chishuiella* | 0.0050 | Module2 | 9 | 0.32 | 0 | 0.97 | 0.1933 | 0 | Peripherals | FTS |
| *Myroides* | 0.0059 | Module2 | 12 | 0.42 | 0.12 | 0.53 | 0.7734 | 0.1528 | Peripherals | FTS |
| *Allorhizobium-Neorhizobium-Pararhizobium-Rhizobium* | 0.0303 | Module2 | 3 | 0.3 | 0 | 1 | -1.5468 | 0 | Peripherals | FTS |
| *Alloprevotella* | 0.0025 | Module3 | 10 | 0.37 | 0.04 | 0.8 | 0.7522 | 0 | Peripherals | FTS |
| *Tyzzerella* | 0.0021 | Module3 | 20 | 0.51 | 0.12 | 0.55 | 0.7522 | 0.545 | Peripherals | FTS |
| *Empedobacter* | 0.0184 | Module2 | 9 | 0.32 | 0 | 0.97 | 0.1933 | 0 | Peripherals | FTS |
| *Eubacterium_hallii_group* | 0.0039 | Module3 | 20 | 0.51 | 0.12 | 0.55 | 0.7522 | 0.545 | Peripherals | FTS |
| *Desulfovibrio* | 0.0032 | Module2 | 3 | 0.3 | 0 | 1 | -1.5468 | 0 | Peripherals | FTS |
| *Chryseobacterium* | 0.0140 | Module3 | 1 | 0.27 | 0 | 0 | -1.9979 | 0 | Peripherals | FTS |
| *Erysipelotrichaceae_UCG-003* | 0.0031 | Module0 | 15 | 0.45 | 0.03 | 0.76 | 0.2582 | 0.48 | Peripherals | FTS |
| *Candidatus_Saccharimonas* | 0.0046 | Module3 | 14 | 0.4 | 0.01 | 0.78 | 0.4466 | 0.4592 | Peripherals | FTS |
| *CAG-352* | 0.0024 | Module0 | 12 | 0.4 | 0.02 | 0.76 | 0.7746 | 0.2778 | Peripherals | FTS |
| *Aeromonas* | 0.0422 | Module1 | 1 | 0.17 | 0 | 0 | -0.7071 | 0 | Peripherals | FTS |
| *Bacteroides* | 0.0390 | Module2 | 8 | 0.37 | 0.03 | 0.64 | -0.6767 | 0.375 | Peripherals | FTS |
| *Parabacteroides* | 0.0025 | Module4 | 1 | 0.02 | 0 | 0 | 0 | 0 | Peripherals | FTS |
| *Roseburia* | 0.0055 | Module3 | 9 | 0.37 | 0 | 1 | 0.4466 | 0 | Peripherals | FTS |
| *Pseudomonas* | 0.0404 | Module2 | 12 | 0.33 | 0.02 | 0.64 | 1.0634 | 0 | Peripherals | FTS |
| *Alistipes* | 0.0039 | Module3 | 12 | 0.43 | 0.05 | 0.67 | 0.4466 | 0.375 | Peripherals | FTS |
| *Fusobacterium* | 0.0120 | Module1 | 2 | 0.21 | 0.04 | 0 | 1.4142 | 0 | Peripherals | FTS |
| *Erysipelatoclostridium* | 0.0026 | Module3 | 12 | 0.43 | 0.05 | 0.67 | 0.4466 | 0.375 | Peripherals | FTS |
| *Eubacterium_coprostanoligenes_group* | 0.0024 | Module0 | 11 | 0.35 | 0.12 | 0.64 | 0.7746 | 0.1653 | Peripherals | FTS |
| *Lachnospiraceae_NK4A136_group* | 0.0174 | Module3 | 14 | 0.4 | 0.01 | 0.78 | 0.4466 | 0.4592 | Peripherals | FTS |
| *Sphingomonas* | 0.0028 | Module3 | 3 | 0.34 | 0.04 | 0.33 | -1.3868 | 0 | Peripherals | FTS |
| *Bacillus* | 0.0394 | Module2 | 9 | 0.32 | 0 | 0.97 | 0.1933 | 0 | Peripherals | FTS |
| *Akkermansia* | 0.0060 | Module4 | 1 | 0.02 | 0 | 0 | 0 | 0 | Peripherals | FTS |
| *Blautia* | 0.0056 | Module0 | 15 | 0.45 | 0.03 | 0.76 | 0.2582 | 0.48 | Peripherals | FTS |
| *Muribaculaceae* | 0.0375 | Module3 | 14 | 0.4 | 0.01 | 0.78 | 0.4466 | 0.4592 | Peripherals | FTS |
| *Deinococcus* | 0.0044 | Module2 | 12 | 0.47 | 0.15 | 0.44 | -0.3867 | 0.5417 | Peripherals | FTS |
| *Luteolibacter* | 0.0023 | Module0 | 12 | 0.5 | 0.21 | 0.52 | -1.291 | 0.6111 | Peripherals | FTS |
| *Flavobacterium* | 0.0190 | Module2 | 5 | 0.3 | 0 | 0.8 | -0.9667 | 0 | Peripherals | FTS |
| *Emticicia* | 0.0031 | Module2 | 13 | 0.42 | 0.07 | 0.6 | 1.0634 | 0.142 | Peripherals | FTS |
| *Acidovorax* | 0.0059 | Module2 | 8 | 0.37 | 0.03 | 0.64 | -0.6767 | 0.375 | Peripherals | FTS |
| *NS11-12_marine_group* | 0.0022 | Module2 | 13 | 0.38 | 0.03 | 0.59 | 1.3534 | 0 | Peripherals | FTS |
| *Bifidobacterium* | 0.0052 | Module0 | 15 | 0.45 | 0.03 | 0.76 | 0.2582 | 0.48 | Peripherals | FTS |
| *Comamonas* | 0.0299 | Module1 | 7 | 0.34 | 0.45 | 0.48 | 0.4913 | 0.4490 | Peripherals | LFS |
| *Acinetobacter* | 0.5280 | Module1 | 1 | 0.2 | 0.00 | 0.00 | -1.4739 | 0.0000 | Peripherals | LFS |
| *Prevotella* | 0.0012 | Module0 | 7 | 0.18 | 0.02 | 0.76 | 0.8321 | 0.0000 | Peripherals | LFS |
| *Eubacterium* | 0.0014 | Module1 | 7 | 0.28 | 0.06 | 0.33 | 1.4739 | 0.0000 | Peripherals | LFS |
| *Lactobacillus* | 0.0070 | Module2 | 7 | 0.21 | 0.01 | 0.67 | -0.7670 | 0.4082 | Peripherals | LFS |
| *Paenibacillus* | 0.0039 | Module3 | 1 | 0.23 | 0.00 | 0.00 | -0.7071 | 0.0000 | Peripherals | LFS |
| *Clostridia_UCG-014* | 0.0006 | Module4 | 2 | 0.23 | 0.00 | 1.00 | -1.4771 | 0.0000 | Peripherals | LFS |
| *Faecalibacterium* | 0.0012 | Module0 | 6 | 0.15 | 0.00 | 0.73 | 0.2774 | 0.0000 | Peripherals | LFS |
| *Elizabethkingia* | 0.0007 | Module3 | 3 | 0.3 | 0.44 | 0.00 | 1.4142 | 0.4444 | Peripherals | LFS |
| *Shewanella* | 0.0037 | Module0 | 5 | 0.15 | 0.00 | 1.00 | -0.2774 | 0.0000 | Peripherals | LFS |
| *Chishuiella* | 0.0007 | Module2 | 8 | 0.24 | 0.01 | 0.79 | 0.6136 | 0.0000 | Peripherals | LFS |
| *Myroides* | 0.0007 | Module2 | 8 | 0.26 | 0.03 | 0.71 | 0.6136 | 0.0000 | Peripherals | LFS |
| *Allorhizobium-Neorhizobium-Pararhizobium-Rhizobium* | 0.0153 | Module0 | 7 | 0.18 | 0.02 | 0.76 | 0.8321 | 0.0000 | Peripherals | LFS |
| *Empedobacter* | 0.0208 | Module0 | 6 | 0.17 | 0.01 | 0.87 | 0.2774 | 0.0000 | Peripherals | LFS |
| *Anaerorhabdus_furcosa_group* | 0.0038 | Module0 | 7 | 0.2 | 0.11 | 0.52 | 0.8321 | 0.0000 | Peripherals | LFS |
| *Desulfovibrio* | 0.0038 | Module1 | 10 | 0.33 | 0.15 | 0.47 | 0.4913 | 0.6200 | Peripherals | LFS |
| *Chryseobacterium* | 0.0661 | Module1 | 3 | 0.23 | 0.00 | 0.33 | -0.4913 | 0.0000 | Peripherals | LFS |
| *Parabacteroides* | 0.0094 | Module2 | 10 | 0.3 | 0.07 | 0.53 | -1.2271 | 0.6600 | Connectors | LFS |
| *dgA-11_gut_group* | 0.0019 | Module1 | 10 | 0.32 | 0.11 | 0.44 | 1.4739 | 0.4600 | Peripherals | LFS |
| *Delftia* | 0.0019 | Module1 | 4 | 0.25 | 0.05 | 0.17 | 0.0000 | 0.0000 | Peripherals | LFS |
| *Bacteroides* | 0.0272 | Module2 | 10 | 0.3 | 0.07 | 0.53 | -1.2271 | 0.6600 | Connectors | LFS |
| *Aeromonas* | 0.0320 | Module1 | 4 | 0.25 | 0.01 | 0.50 | 0.0000 | 0.0000 | Peripherals | LFS |
| *Butyricicoccus* | 0.0008 | Module0 | 4 | 0.2 | 0.00 | 0.83 | -0.8321 | 0.0000 | Peripherals | LFS |
| *Candidatus_Saccharimonas* | 0.0007 | Module2 | 3 | 0.2 | 0.00 | 1.00 | -1.6873 | 0.0000 | Peripherals | LFS |
| *Roseburia* | 0.0012 | Module2 | 10 | 0.25 | 0.03 | 0.58 | 1.0738 | 0.1800 | Peripherals | LFS |
| *Pseudomonas* | 0.0388 | Module1 | 2 | 0.22 | 0.00 | 0.00 | -0.9826 | 0.0000 | Peripherals | LFS |
| *Enterococcus* | 0.0006 | Module0 | 5 | 0.23 | 0.37 | 0.50 | -0.8321 | 0.3200 | Peripherals | LFS |
| *Alistipes* | 0.0011 | Module4 | 6 | 0.24 | 0.02 | 0.47 | -0.6155 | 0.5000 | Peripherals | LFS |
| *Fusobacterium* | 0.0020 | Module0 | 2 | 0.15 | 0.00 | 1.00 | -1.9415 | 0.0000 | Peripherals | LFS |
| *Hydrogenoanaerobacterium* | 0.0006 | Module1 | 10 | 0.32 | 0.11 | 0.44 | 1.4739 | 0.4600 | Peripherals | LFS |
| *Erysipelatoclostridium* | 0.0042 | Module2 | 9 | 0.29 | 0.14 | 0.61 | 0.6136 | 0.1975 | Peripherals | LFS |
| *Eubacterium_coprostanoligenes_group* | 0.0012 | Module1 | 2 | 0.25 | 0.04 | 0.00 | -0.9826 | 0.0000 | Peripherals | LFS |
| *Faecalitalea* | 0.0083 | Module1 | 3 | 0.28 | 0.00 | 0.67 | -0.4913 | 0.0000 | Peripherals | LFS |
| *Lachnospiraceae_NK4A136_group* | 0.0036 | Module2 | 9 | 0.24 | 0.02 | 0.67 | 1.0738 | 0.0000 | Peripherals | LFS |
| *Sphingomonas* | 0.0014 | Module0 | 3 | 0.2 | 0.00 | 1.00 | -1.3868 | 0.0000 | Peripherals | LFS |
| *Bacillus* | 0.0098 | Module2 | 8 | 0.26 | 0.03 | 0.71 | 0.6136 | 0.0000 | Peripherals | LFS |
| *Akkermansia* | 0.0041 | Module4 | 7 | 0.28 | 0.03 | 0.67 | 0.2462 | 0.5714 | Peripherals | LFS |
| *Anaerotruncus* | 0.0006 | Module4 | 6 | 0.28 | 0.07 | 0.47 | 1.9695 | 0.0000 | Peripherals | LFS |
| *Blautia* | 0.0006 | Module0 | 8 | 0.18 | 0.06 | 0.61 | 1.3868 | 0.0000 | Peripherals | LFS |
| *Pedobacter* | 0.0040 | Module3 | 2 | 0.27 | 0.39 | 0.00 | -0.7071 | 0.5000 | Peripherals | LFS |
| *Eubacterium_xylanophilum_group* | 0.0006 | Module2 | 7 | 0.21 | 0.01 | 0.67 | -0.7670 | 0.4082 | Peripherals | LFS |
| *Muribaculaceae* | 0.0065 | Module2 | 9 | 0.24 | 0.02 | 0.67 | 1.0738 | 0.0000 | Peripherals | LFS |
| *Novosphingobium* | 0.0007 | Module4 | 6 | 0.24 | 0.04 | 0.47 | 0.2462 | 0.4444 | Peripherals | LFS |
| *Shinella* | 0.0044 | Module0 | 7 | 0.2 | 0.15 | 0.57 | 0.8321 | 0.0000 | Peripherals | LFS |
| *Luteolibacter* | 0.0231 | Module1 | 1 | 0.2 | 0.00 | 0.00 | -1.4739 | 0.0000 | Peripherals | LFS |
| *Flavobacterium* | 0.0158 | Module4 | 10 | 0.34 | 0.26 | 0.53 | -0.6155 | 0.6600 | Connectors | LFS |
| *Rikenella* | 0.0007 | Module4 | 7 | 0.28 | 0.03 | 0.67 | 0.2462 | 0.5714 | Peripherals | LFS |
| *Acidovorax* | 0.0025 | Module1 | 5 | 0.26 | 0.01 | 0.60 | 0.4913 | 0.0000 | Peripherals | LFS |

| Table S4. Rank sum test at the phylum level | | | | | | | | | | |
| --- | --- | --- | --- | --- | --- | --- | --- | --- | --- | --- |
| Phylum | TS  (Mean) | TS  (Std) | LTS  (Mean) | LTS  (Std) | FTS  (Mean) | FTS  (Std) | LFS  (Mean) | LFS  (Std) | Multigroup  (*P*) | Multigroup  (p-corrected) |
| Deinococcota | 0.0015 | 0.0008 | 0.0055 | 0.0030 | 0.0050 | 0.0025 | 0.0005 | 0.0005 | 0.0007 | 0.0202 |
| Proteobacteria | 0.8020 | 0.0556 | 0.4635 | 0.0877 | 0.4966 | 0.0925 | 0.7043 | 0.1064 | 0.0013 | 0.0202 |
| Synergistota | 0.0000 | 0.0000 | 0.0003 | 0.0001 | 0.0001 | 0.0002 | 0.0000 | 0.0001 | 0.0015 | 0.0202 |
| Patescibacteria | 0.0023 | 0.0022 | 0.0109 | 0.0074 | 0.0066 | 0.0030 | 0.0010 | 0.0011 | 0.0016 | 0.0202 |
| Firmicutes | 0.0945 | 0.0464 | 0.2584 | 0.0503 | 0.2188 | 0.0644 | 0.0904 | 0.0699 | 0.0019 | 0.0202 |
| Verrucomicrobiota | 0.0023 | 0.0012 | 0.0101 | 0.0020 | 0.0113 | 0.0044 | 0.0276 | 0.0206 | 0.0028 | 0.0220 |
| Chloroflexi | 0.0002 | 0.0002 | 0.0060 | 0.0075 | 0.0030 | 0.0027 | 0.0001 | 0.0001 | 0.0034 | 0.0220 |
| Bacteroidota | 0.0783 | 0.0253 | 0.1817 | 0.0248 | 0.1961 | 0.0332 | 0.1615 | 0.0435 | 0.0041 | 0.0220 |
| Halobacterota | 0.0000 | 0.0001 | 0.0013 | 0.0015 | 0.0008 | 0.0007 | 0.0000 | 0.0000 | 0.0042 | 0.0220 |
| Acidobacteriota | 0.0003 | 0.0002 | 0.0041 | 0.0056 | 0.0040 | 0.0051 | 0.0000 | 0.0001 | 0.0044 | 0.0220 |
| Campilobacterota | 0.0008 | 0.0009 | 0.0031 | 0.0009 | 0.0021 | 0.0018 | 0.0004 | 0.0005 | 0.0046 | 0.0220 |
| Nitrospirota | 0.0001 | 0.0001 | 0.0010 | 0.0013 | 0.0003 | 0.0002 | 0.0000 | 0.0000 | 0.0064 | 0.0273 |
| Cyanobacteria | 0.0012 | 0.0011 | 0.0056 | 0.0043 | 0.0041 | 0.0021 | 0.0006 | 0.0005 | 0.0068 | 0.0273 |
| Myxococcota | 0.0000 | 0.0000 | 0.0005 | 0.0006 | 0.0004 | 0.0005 | 0.0000 | 0.0000 | 0.0079 | 0.0294 |
| Planctomycetota | 0.0009 | 0.0012 | 0.0090 | 0.0117 | 0.0039 | 0.0038 | 0.0002 | 0.0002 | 0.0089 | 0.0308 |
| Actinobacteriota | 0.0023 | 0.0023 | 0.0159 | 0.0146 | 0.0155 | 0.0120 | 0.0028 | 0.0016 | 0.0116 | 0.0378 |
| Fusobacteriota | 0.0102 | 0.0083 | 0.0098 | 0.0081 | 0.0214 | 0.0151 | 0.0022 | 0.0032 | 0.0128 | 0.0392 |
| WPS-2 | 0.0000 | 0.0000 | 0.0006 | 0.0010 | 0.0001 | 0.0001 | 0.0000 | 0.0000 | 0.0195 | 0.0528 |
| Modulibacteria | 0.0000 | 0.0000 | 0.0001 | 0.0001 | 0.0000 | 0.0000 | 0.0000 | 0.0000 | 0.0203 | 0.0528 |
| Zixibacteria | 0.0000 | 0.0000 | 0.0001 | 0.0001 | 0.0000 | 0.0000 | 0.0000 | 0.0000 | 0.0203 | 0.0528 |
| Desulfobacterota | 0.0006 | 0.0004 | 0.0049 | 0.0025 | 0.0057 | 0.0039 | 0.0076 | 0.0130 | 0.0231 | 0.0571 |
| Gemmatimonadota | 0.0000 | 0.0000 | 0.0007 | 0.0009 | 0.0009 | 0.0011 | 0.0000 | 0.0000 | 0.0256 | 0.0604 |
| Deferribacterota | 0.0001 | 0.0001 | 0.0003 | 0.0001 | 0.0004 | 0.0002 | 0.0003 | 0.0003 | 0.0397 | 0.0898 |
| Latescibacterota | 0.0000 | 0.0000 | 0.0001 | 0.0001 | 0.0001 | 0.0002 | 0.0000 | 0.0000 | 0.0583 | 0.1264 |
| Caldisericota | 0.0000 | 0.0000 | 0.0000 | 0.0001 | 0.0001 | 0.0001 | 0.0000 | 0.0000 | 0.0855 | 0.1779 |
| FCPU426 | 0.0000 | 0.0000 | 0.0001 | 0.0001 | 0.0000 | 0.0000 | 0.0000 | 0.0000 | 0.0996 | 0.1849 |
| Hydrogenedentes | 0.0000 | 0.0000 | 0.0000 | 0.0000 | 0.0000 | 0.0000 | 0.0000 | 0.0000 | 0.0996 | 0.1849 |
| WS2 | 0.0000 | 0.0000 | 0.0000 | 0.0000 | 0.0000 | 0.0000 | 0.0000 | 0.0000 | 0.0996 | 0.1849 |
| Spirochaetota | 0.0004 | 0.0005 | 0.0010 | 0.0007 | 0.0003 | 0.0002 | 0.0004 | 0.0007 | 0.1957 | 0.3509 |
| Nanoarchaeota | 0.0000 | 0.0000 | 0.0000 | 0.0001 | 0.0000 | 0.0000 | 0.0000 | 0.0000 | 0.2063 | 0.3575 |
| Armatimonadota | 0.0000 | 0.0000 | 0.0001 | 0.0002 | 0.0001 | 0.0002 | 0.0000 | 0.0000 | 0.2292 | 0.3703 |
| Abditibacteriota | 0.0000 | 0.0000 | 0.0001 | 0.0002 | 0.0000 | 0.0000 | 0.0000 | 0.0000 | 0.2350 | 0.3703 |
| Sumerlaeota | 0.0000 | 0.0000 | 0.0001 | 0.0001 | 0.0000 | 0.0000 | 0.0000 | 0.0000 | 0.2350 | 0.3703 |
| Elusimicrobiota | 0.0000 | 0.0000 | 0.0001 | 0.0002 | 0.0001 | 0.0002 | 0.0000 | 0.0000 | 0.2620 | 0.3785 |
| NB1-j | 0.0000 | 0.0000 | 0.0000 | 0.0001 | 0.0000 | 0.0001 | 0.0000 | 0.0000 | 0.2620 | 0.3785 |
| SAR324_cladeMarine_group_B | 0.0000 | 0.0001 | 0.0001 | 0.0001 | 0.0000 | 0.0000 | 0.0000 | 0.0000 | 0.2620 | 0.3785 |
| Methylomirabilota | 0.0000 | 0.0000 | 0.0001 | 0.0002 | 0.0001 | 0.0001 | 0.0000 | 0.0000 | 0.2871 | 0.4034 |
| Acetothermia | 0.0000 | 0.0000 | 0.0000 | 0.0000 | 0.0000 | 0.0000 | 0.0000 | 0.0000 | 0.3916 | 0.4736 |
| Cloacimonadota | 0.0000 | 0.0000 | 0.0001 | 0.0001 | 0.0000 | 0.0000 | 0.0000 | 0.0000 | 0.3916 | 0.4736 |
| Entotheonellaeota | 0.0000 | 0.0000 | 0.0000 | 0.0000 | 0.0000 | 0.0000 | 0.0000 | 0.0000 | 0.3916 | 0.4736 |
| Fermentibacterota | 0.0000 | 0.0000 | 0.0000 | 0.0000 | 0.0000 | 0.0000 | 0.0000 | 0.0000 | 0.3916 | 0.4736 |
| MBNT15 | 0.0000 | 0.0000 | 0.0000 | 0.0000 | 0.0000 | 0.0000 | 0.0000 | 0.0000 | 0.3916 | 0.4736 |
| Thermoplasmatota | 0.0000 | 0.0000 | 0.0000 | 0.0000 | 0.0000 | 0.0000 | 0.0000 | 0.0000 | 0.3916 | 0.4736 |
| Euryarchaeota | 0.0000 | 0.0000 | 0.0000 | 0.0000 | 0.0000 | 0.0001 | 0.0000 | 0.0000 | 0.4383 | 0.5180 |
| Crenarchaeota | 0.0000 | 0.0000 | 0.0000 | 0.0000 | 0.0000 | 0.0000 | 0.0000 | 0.0000 | 0.5131 | 0.5752 |
| Aenigmarchaeota | 0.0000 | 0.0000 | 0.0000 | 0.0001 | 0.0000 | 0.0000 | 0.0000 | 0.0000 | 0.5531 | 0.5752 |
| Fibrobacterota | 0.0000 | 0.0000 | 0.0000 | 0.0001 | 0.0000 | 0.0000 | 0.0000 | 0.0000 | 0.5531 | 0.5752 |
| Nitrospinota | 0.0000 | 0.0000 | 0.0000 | 0.0001 | 0.0000 | 0.0001 | 0.0000 | 0.0000 | 0.5531 | 0.5752 |
| RCP2-54 | 0.0000 | 0.0000 | 0.0000 | 0.0000 | 0.0000 | 0.0000 | 0.0000 | 0.0000 | 0.5531 | 0.5752 |
| Sva0485 | 0.0000 | 0.0000 | 0.0000 | 0.0000 | 0.0000 | 0.0000 | 0.0000 | 0.0000 | 0.5531 | 0.5752 |
| Dependentiae | 0.0000 | 0.0000 | 0.0000 | 0.0000 | 0.0000 | 0.0000 | 0.0000 | 0.0000 | 0.5663 | 0.5774 |
| Bdellovibrionota | 0.0014 | 0.0028 | 0.0004 | 0.0004 | 0.0003 | 0.0003 | 0.0002 | 0.0002 | 0.9262 | 0.9262 |

| Table S5 The results of Tukey's post hoc multiple comparison test. | | | | | | | | |
| --- | --- | --- | --- | --- | --- | --- | --- | --- |
| Groups | Q_value | | | | P_value | | | |
|  | ACE | Chao1 | Shannon | Simpson | ACE | Chao1 | Shannon | Simpson |
| LTS vs TS | 5.20063 | 5.22965 | 11.58465 | 10.70963 | 0.00749 | 0.00716 | <0.0001 | <0.0001 |
| FTS vs TS | 5.4413 | 5.47133 | 10.15107 | 9.85659 | 0.00511 | 0.00487 | <0.0001 | <0.0001 |
| FTS VS LTS | 0.24067 | 0.24167 | 1.43358 | 0.85304 | 0.99819 | 0.99816 | 0.74343 | 0.92986 |
| LFS vs TS | 0.10351 | 0.10466 | 0.48741 | 0.45984 | 0.99985 | 0.99985 | 0.98551 | 0.98776 |
| LFS vs LTS | 5.30414 | 5.33431 | 11.09724 | 10.24979 | 0.00636 | 0.00606 | <0.0001 | <0.0001 |
| LFS vs FTS | 5.5448 | 5.57598 | 9.66366 | 9.39675 | 0.00433 | 0.00412 | <0.0001 | <0.0001 |

Note: *P<*0.05 indicates a statistically significant difference between the two groups.

**Fig. S1** Rarefaction curves (A), Shannon index curves (B), Rank abundance curves (C), and Species accumulation curves (D)


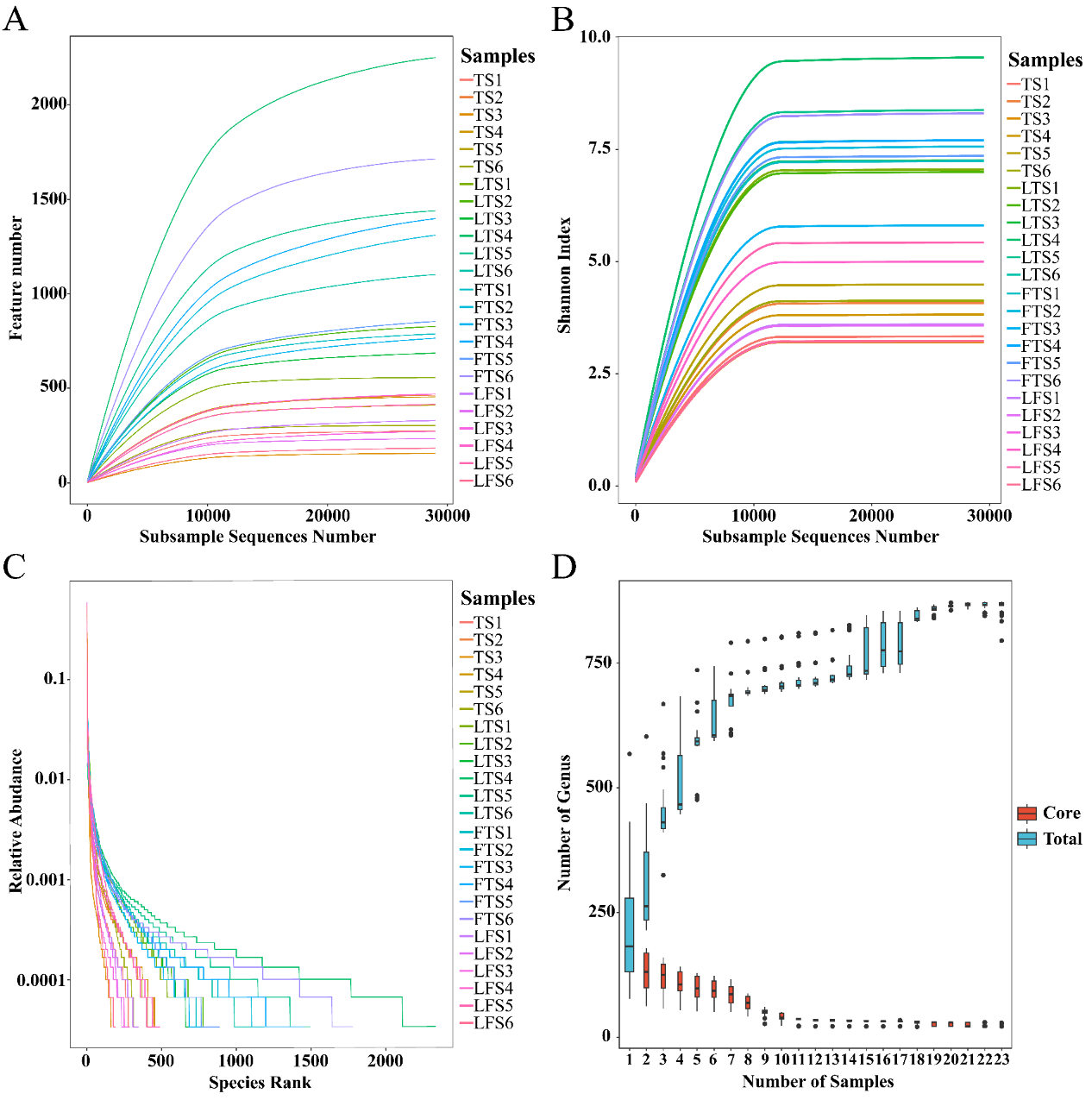

Supplement: Supplementary file 1 [file Table_1.docx]
